# A Real-Time Quantitative PCR Method Specific for Detection and Quantification of the First Commercialized Genome-Edited Plant

**DOI:** 10.3390/foods9091245

**Published:** 2020-09-07

**Authors:** Pradheep Chhalliyil, Heini Ilves, Sergei A. Kazakov, Stephanie J. Howard, Brian H. Johnston, John Fagan

**Affiliations:** 1 Health Research Institute, 505 Dimick Drive, P.O. Box 370, Fairfield, IA 52556, USA; pradheep@hrilabs.org; 2 Somagenics, Inc., 2161 Delaware Ave, Suite E, Santa Cruz, CA 95060, USA; hilves@somagenics.com (H.I.); skazakov@somagenics.com (S.A.K.); bjohnston@somagenics.com (B.H.J.); 3 The Sustainability Council of New Zealand, P.O. Box 24304, Wellington 6142, New Zealand; stephanie.howard@sustainabilitynz.org

**Keywords:** GMO detection, GMO quantitation, genome-edited crops, real-time quantitative PCR, regulatory enforcement, biosafety, traceability

## Abstract

Discussion regarding the regulatory status of genome-edited crops has focused on precision of editing and on doubts regarding the feasibility of analytical monitoring compliant with existing GMO regulations. Effective detection methods are important, both for regulatory enforcement and traceability in case of biosafety, environmental or socio-economic impacts. Here, we approach the analysis question for the first time in the laboratory and report the successful development of a quantitative PCR detection method for the first commercialized genome-edited crop, a canola with a single base pair edit conferring herbicide tolerance. The method is highly sensitive and specific (quantification limit, 0.05%), compatible with the standards of practice, equipment and expertise typical in GMO laboratories, and readily integrable into their analytical workflows, including use of the matrix approach. The method, validated by an independent laboratory, meets all legal requirements for GMO analytical methods in jurisdictions such as the EU, is consistent with ISO17025 accreditation standards and has been placed in the public domain. Having developed a qPCR method for the most challenging class of genome edits, single-nucleotide variants, this research suggests that qPCR-based method development may be applicable to virtually any genome-edited organism. This advance resolves doubts regarding the feasibility of extending the regulatory approach currently employed for recombinant DNA-based GMOs to genome-edited organisms.

## 1. Introduction

In recent years, biotechnologists have begun to employ genome editing methods such as ODM (oligonucleotide-directed mutagenesis), CRISPR/Cas (clustered regularly interspaced short palindromic repeats/CRISPR associated protein), TALEN (transcription activator-like effector nuclease) and ZFN (zinc finger nuclease) to modify the characteristics of organisms important in production of food and feed. At present, two genome-edited plants have been commercialized in North America, a herbicide-tolerant canola variety [1] and a soy bean variety with modified oil composition [2].

The regulatory landscape regarding genome-edited crops is currently not well defined globally. Despite the commercialization of the above two genome-edited crops and recent policy changes by the US Department of Agriculture, the US has not formalized a comprehensive position on the regulation of genome editing methods. A handful of other countries have established laws or regulations regarding genome-edited plants [3]. The European Court of Justice has ruled [4] that crops modified by directed mutagenesis fall within the scope of Directive 2001/18/EC on the release of GMOs into the environment [5]. In practice, this means that genome-edited crops are regulated according to that Directive [5]. Preceding and following this decision, there has been sustained discussion with wide-ranging perspectives regarding the regulation of these methods among EU [6,7,8] and member state [9,10,11,12] government representatives, within the Convention on Biodiversity [13,14], and among representatives of the biotechnology industry [15,16] and the academic community [3,17,18,19,20,21,22,23].

Although the precision of genome editing and range of off-target and other unintended effects, especially in comparison with random mutagenesis, have been one focus of the discussion regarding regulatory status of genome-edited organisms [3,19,22,23,24,25], a second, prominent focus has been feasibility of developing methods for detecting genome-edited organisms [3,7,10,25,26,27,28,29]. One of the arguments put forward to justify regulating genome-edited crops differently from recombinant DNA-based GMOs is that Directive 2001/18/EC requires analysis-based surveillance of GMOs, while, it is claimed, there are many technical and regulatory challenges that make development of GMO regulation-compliant analytical identification and quantitation methods for genome-edited organisms difficult or even impossible [7,10,25,26,28,29]. However, these claims are controversial [3,27].

To date, discussions regarding detection and quantification of genome-edited organisms have remained mostly on the theoretical level [28]. To provide an empirical basis for the discussion, we have begun to explore these questions in the laboratory and have successfully developed a real-time quantitative PCR (qPCR) method for identification and quantitation of the first genome-edited crop commercialized, an herbicide-tolerant canola. This method is fully compliant with regulations for monitoring of GMOs in the EU and similar jurisdictions. A single base pair edit in the canola *AHAS1C* gene has rendered the resulting gene-product tolerant to sulfonylurea and imidazolinone herbicides [30]. We deemed this product a worthy test of the question of whether methods could be developed for identification and quantitation of genome-edited crops, since this product embodies many of the more challenging features that genome-edited crops may present, such as the fact that: (1) the edit consists of a single base pair modification and, (2) while the edit has been carried out on one member of a multigene family (*AHAS1C*), (3) a second member of the family (*AHAS3A*) carries the same single base pair modification generated via chemical mutagenesis. Thus, it was necessary to design a method capable of distinguishing between these two.

Using standard qPCR methodology, in conjunction with the use of locked nucleic acids (LNAs) in primer design, we were able to develop a high-sensitivity quantitative detection method for the single-nucleotide genome edit described above. This approach is likely to apply to other single-nucleotide edits and, since the scientific community has two decades of experience successfully using standard qPCR methods to quantitatively detect indels and gene insertions, this is an approach to method development that may be applicable to all classes of genome-edited crop for which at least minimal construct information is available. This is an important conclusion, because it establishes a clear path forward for analysis-based regulation of genome-edited organisms that is fully compliant with current GMO regulations in jurisdictions such as the EU [5,31,32] and fully consistent with existing practices, workflows, and expertise available in contemporary GMO analytical laboratories. This paper describes the development and independent validation of this method, which has been placed in the public domain, available for use by all laboratories.

## 2. Materials and Methods

### 2.1. Canola Germplasm

Seed samples of 20 wild-type canola varieties were obtained through the U.S. National Plant Germplasm System (North Central Regional Plant Introduction Station, Ames, IA, USA). The specific varieties are listed in Table S1 in Supplementary Materials. They include varieties bred for production in Bangladesh, Canada, China, Denmark, France, Germany, Japan, the Netherlands, New Zealand, Poland, Russia, South Korea, Sweden, and the United States. Commercial seed from three Clearfield sulfonylurea and imidazolinone herbicide-tolerant canola varieties, developed via chemical mutagenesis, were used, 5545 CL (Brett-Young Seeds Ltd., Winnipeg, MB, Canada), CS2200 CL (Canterra Seeds LTD., Winnipeg, MB, Canada), and 2022 CL (Viterra Inc., Calgary, AB, Canada). Certified seed from three SU (sulfonylurea and imidazolinone herbicide-tolerant) canola varieties, C1511, C5507, and 40K (Cibus US LLC/Falco Brand, San Diego, CA, USA) all developed by oligonucleotide directed mutagenesis [1,30] were used.

### 2.2. Sanger Sequencing

Sanger sequencing of a segment of the *AHAS1C* gene that included two single-nucleotide variants (SNVs) unique to *AHAS1C* and the genome edit at position 1676, which is common to both *AHAS1C* and *AHAS3A*, was used to characterize the three SU canola varieties and the three Clearfield varieties described in Section 2.1. For Sanger sequencing, 10 ng of canola genomic DNA was amplified with *AHAS1C* gene-specific primers in a volume of 50 microliters, using 5 units of HotFirepol (Solis BioDyne, Tartu, Estonia). The forward primer was CTATTGCTGCAGTTTCCCAAC, spanning base pairs 284 to 263 upstream of the start codon of the *AHAS1C* gene; this primer is selective for the *AHAS1C* gene versus the *AHAS3A* gene. The reverse primer was GGTCCCCGAGATAAGTGTGAGCTCTG, spanning base pairs 1706 to 1726 of the *AHAS1C* gene. PCR profile: 1 × 95 °C for 15 min, 40 × (95 °C for 30 s, 65 °C for 30 s, 72 °C for 60 s), 1 × 72 °C for 10 min. The expected PCR product, 2007 bp long, was purified from the agarose gel using Zymoclean Gel DNA Recovery kit (ZymoResearch, Irvine, CA, USA). A total of 100 ng of PCR product was subjected to Sanger sequencing at Retrogen (San Diego, CA, USA) using the primer CTCCGCAGTACGCGATTCAGA, which spans base pairs 1343 through 1363 of the *AHAS1C* gene. The length of the read was 384 nucleotides. All primers were designed based on the *AHAS1C* sequence, Genbank accession number Z11524.1. All primers were synthesized at IDT (Cedar Rapids, IA, USA).

### 2.3. DNA Extraction

Genomic DNA was extracted from ground seeds using the DNeasy Plant Mini Kit (Qiagen, Hilden, Germany, cat. no. 69104). The extracted DNA was further purified from degraded DNA or other contaminants by passing through an Illustra MicroSpin G-50 column (cat. no. 27533001). The concentrations and quality of the extracted DNA solutions were evaluated by measuring UV absorbance (ND-1000, NanoDrop Technologies, Wilmington, DE, USA). All genomic DNA solutions were adjusted to concentrations of 30 and 10 ng/μL for real-time PCR analyses. DNA solutions were stored at −20 °C until used.

### 2.4. Oligonucleotide Primers and Probes

DNA primers and TaqMan^®^ probes were synthesized by Integrated DNA Technologies, Inc. (Coralville, IA, USA), except for the locked nucleic acid (LNA)-containing primer, which was obtained from QIAGEN Genomic Services (Frederick, MD, USA). The probes were labeled with 6-carbocyfluorescein (FAM) at the 5′ end and BHQ-1 at the 3′ end.

### 2.5. Real-Time Quantitative PCR

Real-time quantitative PCR assays were performed using an Applied Biosystems 7500 Fast qPCR system (Thermo Fisher Scientific) in a final volume of 25 μL containing 300 ng of a DNA template, 12.5 μL Master Mix (Kapa Probe Fast), 0.4 μL of each primer (100 μM), and 0.2 μL probe (100 μM) (SU canola method) and 0.2 μL of each primer (100 μM), and 0.1 μL probe (100 μM) (*CruA* method). The step-cycle program was 10 min at 95 °C, followed by 45 cycles of 30 s at 95 °C, and 1 min at 60 °C.

### 2.6. Independent Method Validation

The SU canola-specific qPCR method was subjected to formal validation by the Laboratory for GMO Analysis (LGA) of the Umweltbundesamt GmbH (Environment Agency Austria), which is accredited according to ISO 17025:2017 for GMO analysis and is a member of the European Network of GMO Laboratories (ENGL). The validation was conducted with regard to LOD/POD and robustness according to the “Guidelines for the single-laboratory validation of qualitative real-time PCR methods” of the German Federal Office of Consumer Protection and Food Safety [Bundesamt für Verbraucherschutz und Lebensmittelsicherheit (BVL)] [33] as well as the ENGL guidance document, “Verification of analytical methods for GMO testing when implementing interlaboratory validated methods-Version 2” [34].

The methods used by LGA in the validation were as described in the Methods section of this paper except for a few minor differences: (1) LGA used a denaturation time of 15 s and we (HRI/SomaGenics) used 30 s. This difference would only become significant if the amplicon size were 2 kb or greater. (2) We used Kapa Probe Fast qPCR master mix, while LGA used Kapa Probe Force qPCR master mix. The polymerase used in the latter master mix is considered more resistant to inhibitory contaminants that might be present in the DNA preparation. However, this difference in master mixes is not significant since both laboratories used very stringent DNA purification procedures that differed only in that LGA used Illustra MicroSpin S300 columns (cat no. 27533001) as a cleanup step while we used Illustra MicroSpin G-50 columns. (3) For the *CruA* probe, LGA used TAMRA as quencher, while we used BHQ-1. These two quenchers perform quite similarly. These minor differences would not be expected to influence the performance of the method, which is confirmed by the consistency of results obtained by the two laboratories.

### 2.7. Intellectual Property

Consistent with the commitment to open source the detection method described in this article, we have prepared a statement regarding the associated intellectual property, which can be found as File S1. Statement Regarding Associated Intellectual Property of this paper.

## 3. Results

### 3.1. Development of a qPCR Method Specific for SU Canola

According to the literature, the canola *AHAS* gene family consists of five genes. *AHAS1C* and *AHAS3A* encode catalytic subunits of the functional acetohydroxy acid synthase. *AHAS4A* and *AHAS5C* are probably inactive, appearing to have interrupted coding sequences. *AHAS2A* appears to have a separate function and differs in sequence from *AHAS1C* and *AHAS3A* in the coding region, the signal peptide region and in upstream DNA sequences [35]. Whereas *AHAS1C* and *AHAS3A* are constitutively expressed, *AHAS2A* is expressed in specialized ovule tissues [36]. *AHAS1C* and *AHAS5C* reside on the C genome, while the other three genes reside on the A genome of *Brassica napus* [35].

**Figure 1:**
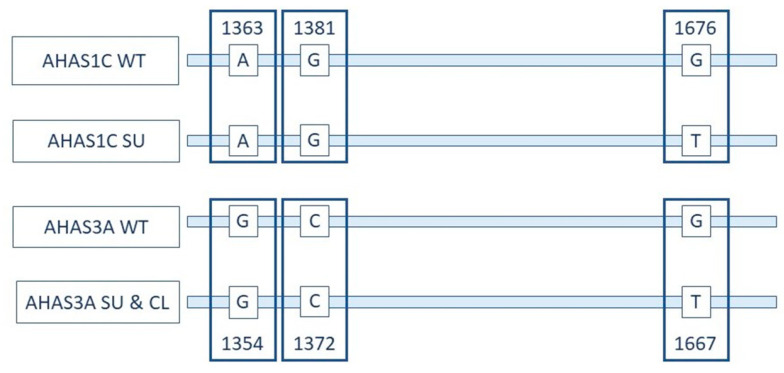
Relationship between *AHAS1C* and *AHAS3A* and positions of a key mutation. Representation of coding strands of the *AHAS1C* gene and *AHAS3A* gene in wild-type (WT), genome edited for sulfonylurea and imidazolinone tolerance (SU) and Clearfield (CL) canola varieties. These regions of the *AHAS1C* and *AHAS3A* genes are identical except for the bases indicated. Differences in numbering of the two genes is due to differences in sequences upstream of the region depicted. The two SNVs at 1363/1354 and at 1381/1372 differentiate *AHAS1C* from *AHAS3A*. The SNV at 1676/1667 differentiates *AHAS1C SU*, *AHAS3A SU* and *AHAS3A CL* from *AHAS1C WT* and *AHAS3A WT*. See papers cited in this section for details on structure of the *AHAS* gene family.

The 1300 to 1700 bp regions of the *AHAS1C* and *AHAS3A* genes are diagramed in Figure 1. The G-to-T SNV at 1676/1667 confers both sulfonylurea and imidazolinone herbicide tolerance. This SNV is shared by *AHAS1C SU* and *AHAS3A*, while the two SNVs at 1363/1354 and 1381/1372 are shared by *AHAS1C* SU and *AHAS1C* wild type (WT).

Canola event BnALS-57 was generated by oligonucleotide directed G-to-T mutagenesis at base pair 1676 of the *AHAS1C* gene. Variety 5715 was created by crossing canola BnALS-57 with the commercial Clearfield canola variety SP Cougar CL [30,37,38], in which a G-to-T mutation at position 1667 of the *AHAS3A* gene was created by chemical mutagenesis, conferring herbicide tolerance on the *AHAS3A* gene product. The result was a canola variety in which both the *AHAS3A* and *AHAS1C* genes carried mutations conferring tolerance to both sulfonylurea and imidazolinone herbicides, *AHAS3A* (via chemical mutagenesis) and *AHAS1C* (via ODM). Based on the fact that variety 5715 was the only Cibus ODM variety authorized/deregulated at the time varieties C1511, C5507 and 40K were commercialized, it can be concluded that these varieties were derived from variety 5715.

**Figure 2:**
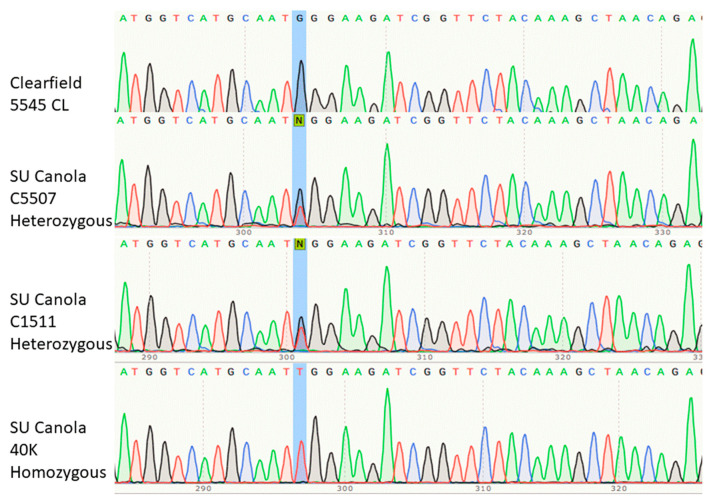
Portion of the Sanger sequencing of the segment of the *AHAS1C* gene spanning the site of genome editing, at position 1676, highlighted in blue. This base is homozygous G (wild type) in Clearfield canola variety 5545 CL, homozygous T (mutant) in SU canola variety 40K and G/T heterozygous in SU canola varieties C5507 and C1511. The region sequenced extended from *AHAS1C*-specific SNVs at positions 1363 and 1381 to beyond position 1704, verifying the location of the genome edit at position 1676. The location of the primer used for sequencing is indicated in Figure 3.

We verified that the configuration depicted in Figure 1 was present in SU canola varieties 40K, C1511 and C5507 by sequencing the *AHAS1C* region from 1340 to 1700 by the Sanger method. The results of the Sanger sequencing were confirmed by comparison with the *AHAS*1C sequence as reported in GenBank Accession Z11524.1 and the description of the genome-edited *AHAS1C* gene [33,34,39]. The accuracy of the Sanger sequencing results is also confirmed by the ability of the primers designed on the basis of the Sanger sequencing to discriminate between SU canola and Clearfield canola. As shown in Figure 2, the nucleotide found at position 1676 in Clearfield canola variety 5545 CL was G (highlighted in blue), which is the wild type according to the sequence of *Brassica napus* found in GenBank (accession Z11524.1). In contrast, the nucleotide at position 1676 in SU canola variety 40K was found to be T, which is the SNV that confers tolerance to sulfonylurea and imidazolinone herbicides [30,37,38]. Interestingly, both T and G were found to be present at position 1676 in varieties C1511 and C5507. Although there is no confirmation of this in any publication from Cibus, this suggests that these two varieties are heterozygous, while 40K is homozygous at position 1676. We conclude that all three varieties, 40K, C1511 and C5507 possess the characteristics expected for SU canola, and that canola variety 5545 CL possesses the characteristics expected for a Clearfield canola variety. Full data for Sanger sequencing are presented in Supplementary Materials Table S2. Similar Sanger sequencing results were found for the two other Clearfield varieties, CS2200 CL and 2022 CL.

**Figure 3:**
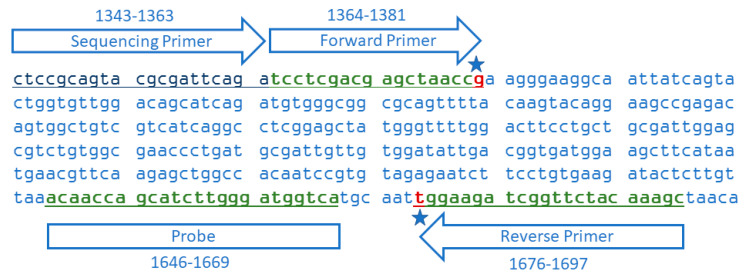
Sequence of part of exon 2 of the SU canola *AHAS1C* gene showing alignments of the primers and probe for the 334 bp amplicon. The forward primer and probe correspond to the target sequence while the reverse primer is complementary to the target sequence. The star by the forward primer indicates the G that is characteristic of the *AHAS*1C gene, distinguishing it from the *AHAS*3A gene which has a C at that site. The star on the reverse primer indicates the single SNV that confers herbicide tolerance on *AHAS*1C SU and *AHAS*3A SU and CL. The sequencing primer is the oligonucleotide use as primer for the Sanger sequencing presented in Figure 2.

To uniquely distinguish *AHAS1C* SU from *AHAS3A* SU and *AHAS3A* CL, we developed a forward primer that targeted the *AHAS1C* SNV at 1381, and a reverse primer that targeted the *AHAS1C* SNV at 1676, as is illustrated in Figure 3. PCR amplification using these primers generated a 334 base pair amplicon specific for *AHAS1C* SU. The sequence of these primers and the corresponding probe are delineated in Table 1, along with sequences of primers and probe for a taxon-specific endogenous reference gene, which generates a 101 bp amplicon of the cruciferin A gene (*CruA*) [39].

### 3.2. Specificity of the SU Canola-Specific qPCR Method

**Figure 4:**
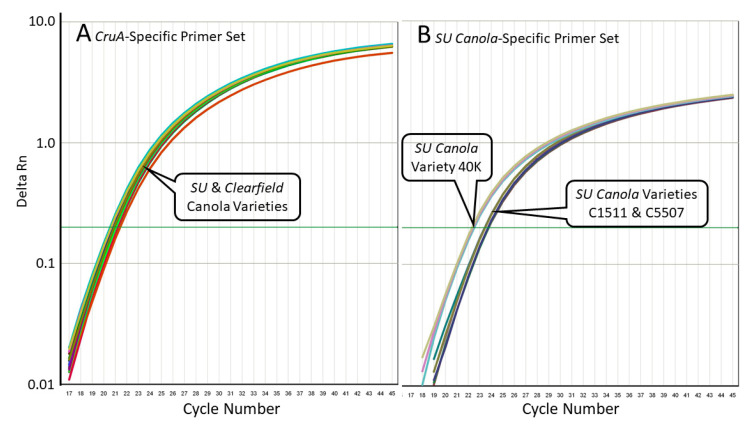
Quantitative PCR amplification curves for the results shown in Table 2, with primers specific for the *CruA* gene (Panel (**A**)) or with primers specific for the SU canola *AHAS1C-SU* gene (Panel (**B**)). In Panel (**B**), amplification occurred only with the SU canola varieties 40K, C1511, and C5507, while in Panel (**A**), amplification also occurred with the Clearfield canola varieties.

We evaluated the specificity of these primer sets using DNA isolated from Clearfield canola varieties that carry the G-to-T mutation at position 1667 in the *AHAS3A* gene, but not in the *AHAS1C* gene and from SU canola DNA, which carries the G-to-T mutation at position 1667 in the *AHAS*3A gene and at position 1676 in the *AHAS*1C gene. As shown in Table 2 and Figure 4, while the *CruA* PCR system amplified both Clearfield and SU canola DNA, the SU canola-specific PCR system amplified only the DNA from SU canola. There was no amplification of the water/no-template control. As expected, since C5507 and C1511 appear to be heterozygous based on Sanger sequencing, while 40K appears to be homozygous (see Figure 2), the Ct for 40K with the SU canola primer-probe set was roughly 1 Ct lower than that of C1511 and C5507. See Table S3 Supplementary Materials for original data.

To confirm the specificities of the *AHAS1C-SU* PCR system for SU canola, we tested its ability to amplify *AHAS1C* sequences from 20 different wild-type canola varieties. Whereas the primer set targeting the *CruA* canola reference gene amplified DNA from all 20 wild-type varieties as well as DNA from the three SU canola varieties and a Clearfield canola variety (Figure 4A), the SU canola-specific primer set failed to amplify DNA from any of the 20 varieties of wild-type canola or from the Clearfield variety but did amplify all three of the SU canola DNA positive controls (Figure 4B). Weak background amplification was observed in a few reactions of two of the wild-type varieties, but this was not consistent among replicates. Of the 93 replicates run with wild-type or Clearfield canola, only 3 showed amplification, and this occurred only at very high cycle numbers, 41 or greater, in contrast to a Ct of 25.3 for an equivalent concentration of SU canola DNA. See Table S3 (Clearfield) and Table S4 (wild-type) in Supplementary Materials for original data, which shows lack of consistency among replicates. Weak background was also seen for the no DNA control for the *CruA* but not the *AHAS*1c SU primer set. For *Cru*A, the difference in Ct between the no DNA sample and samples containing 300 ng DNA was 14.9, which clearly differentiates background from real signals.

**Figure 5:**
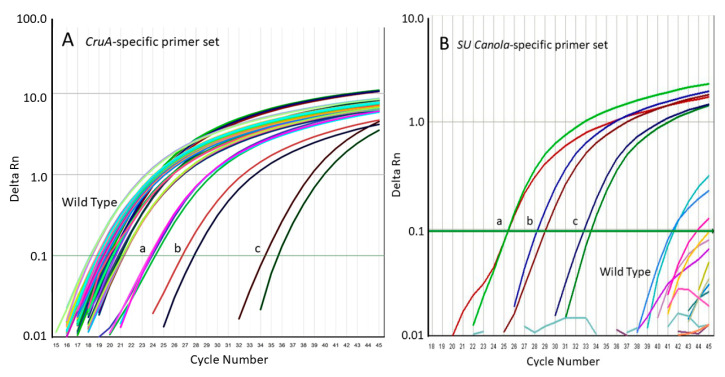
Quantitative PCR amplification curves for the results shown in Table 3. Panel (**A**), amplification using the qPCR method specific for the canola endogenous reference gene (*CruA*); Panel (**B**), amplification using the qPCR method specific for the *AHAS1C-SU* SNV of SU canola. Positive controls are DNA from SU canola event 40K at 300 (a), 30 (b), and 3 (c) ng 40K DNA per reaction.

The 20 wild-type varieties tested are representative of canola varieties in production globally, including varieties from Bangladesh, Canada, China, Denmark, France, Germany, Japan, The Netherlands, New Zealand, Poland, Russia, South Korea, Sweden, and the United States. See Supplementary Materials Table S1 for accession numbers and the origins of all varieties used. From the results presented in Table 2 and Table 3 and Figure 4 and Figure 5, we conclude that the SU canola-specific qPCR method is highly selective for SU canola and does not detect the wild-type or Clearfield canola varieties tested.

Based on the above work, when low level presence of GM material can be expected, the following protocol provides a consistent and standardized approach for routine surveillance testing, consistent with regulatory norms of the EU and other jurisdictions, for determining whether SU canola has been detected with the *AHAS1C-SU* PCR system. 

**Figure 6:**
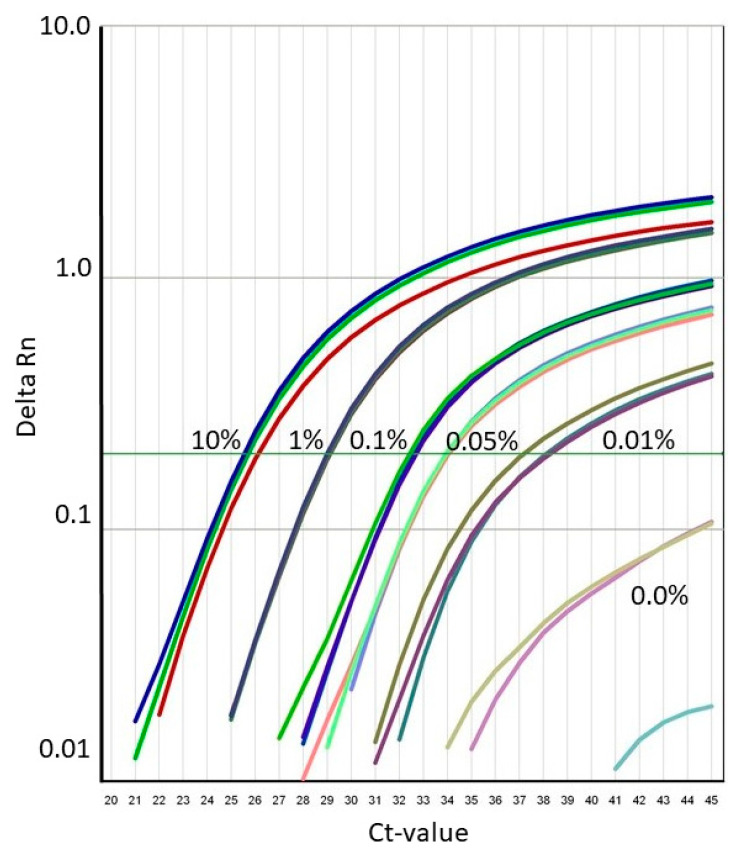
Typical amplification curves for the results shown in Table 3 of SU canola DNA variety 40K mixed at five concentrations with Clearfield canola DNA variety 5545 CL.

First, as specified [40,41], in most cases the amplification threshold is set at 10-times the standard deviation of the baseline fluorescence. To establish the baseline fluorescence value and determine the standard deviation, the baseline fluorescence is sampled from Ct = 3 to two Cts preceding the Ct of the most abundant sample in the run [40,41] This corresponds to the default or auto setting for the threshold in most PCR systems. Second, amplification profiles with Ct values less than 32 are categorized as specific detection of SU canola. Third, amplification profiles with Ct values of 38 or greater are categorized as non-specific amplification. In this case, amplifications observed are considered background noise from quantitative real-time PCR chemistry. Fourth, amplification profiles with Ct values ranging from 32 up to 38 shall be categorized as inconclusive and require confirmation before being declared positive or negative for the presence of SU canola. As is apparent from Figure 6, a Ct of 32 corresponds roughly to the limit of detection of the *AHAS1C-SU* PCR system, established by the LGA (data discussed below and presented in detail in Supplementary Materials, Item 6). Fifth, when such an amplification profile is observed, the sample should be rerun in 12 replicates. Only if all 12 replicates are positive should the result be considered a candidate positive. These criteria are consistent with the specifications for determining the limit of detection set out in the ENGL’s Definition of Minimum Performance Requirements for Analytical Methods of GMO Testing [42].

For final confirmation, in cases where all 12 replicates yield amplification profiles, the amplicons from two of the positive replicates of the candidate positive sample should be subjected to Sanger sequencing, essentially as described in Section 2.2 of Methods, except using the *AHAS1C-SU* PCR primers. Only if the sequence of the amplicon is confirmed to match that of *AHAS1C-SU* would the sample be declared as positive for SU canola. This sequence confirmation is necessary to establish a legally defensible basis for declaring such weak PCR amplifications as definitively indicating the presence of SU canola. In cases where the Sanger sequencing does not match the *AHAS1C-SU* sequence, the sequence data can be used to design a qPCR test, which can be used in the context of the matrix approach, to routinely distinguish that canola variant from SU canola.

### 3.3. Precision, Trueness, Limit of Quantitation and Limit of Detection of the SU Canola Method

To determine the precision, trueness, limit of quantitation and limit of detection of the SU canola method, we combined decreasing proportions of DNA from SU canola event 40K with DNA from Clearfield canola variety 5545 CL. The units of relative concentration of SU canola DNA in 300 ng total DNA per reaction are weight SU canola DNA/weight total DNA (SU canola plus canola variety 5545 CL) percent. The results for proportions of SU canola DNA of 10%, 1.0%, 0.5%, 0.1%. 0.05% 0.01% and 0.0% are shown in Table 4 and Figure 6. Total DNA was maintained constant at 300 ng/reaction, and reaction conditions were as described in Methods. Twelve replicate amplification reactions were carried out with each DNA mixture to assess the precision and trueness of amplification of the SU canola-specific PCR method. At 0.05% SU canola DNA, the relative standard deviation among replicates was ±16.8% and the percent trueness was ±6.93%. Both precision and trueness comply with ENGL acceptance criteria (relative standard deviation ≤ 25%, trueness ≤ ±25%) [42]. Based on these results, we conclude that the relative limit of quantitation for the SU canola PCR method is 0.05% for SU canola DNA in 300 ng total DNA. From this, we extrapolated the relative limit of detection to be 0.025% for SU canola DNA in 300 ng total DNA. See Supplementary Materials Table S5a,b for details of LOD and LOQ determinations.

### 3.4. Independent Validation of the SU Canola-Specific qPCR Method

Independent validation of the SU canola-specific qPCR method was conducted by the Laboratory for GMO Analysis (LGA) of the Umweltbundesamt GmbH (Environment Agency Austria). Their results are summarized below. The full report is provided in Supplementary Materials, Item 6.

The LGA determined the absolute limit of detection by two distinct methods, concluding that the LOD was 5 to 10 genomic copies with a level of confidence of 95%, which is in line with the ENGL acceptance criteria of <25 copies with a level of confidence of 95% [42]. This is consistent with the relative LOD determined during method development.

Specificity of the SU canola-specific qPCR method was assessed by the LGA by performing tests with DNA from eight wild-type canola varieties, three Clearfield varieties, six GM canola varieties, and DNA from corn, soy, rice, potato, and cotton. The SU canola-specific primer set failed to amplify all of these, while consistently amplifying the two SU canola varieties.

As part of the validation, LGA verified that the method met robustness criteria for six key factors, including PCR equipment, PCR master mix, annealing temperature, volume variation, primer concentration, and probe concentration.

The validation also verified quantitative performance from 0.10% to 5.00% SU canola DNA, and PCR efficiency and linearity were also verified over the full range of quantitation. In parallel, trueness and precision were assessed at 0.10%, 1.00% and 5.00% SU canola DNA. Both were well within the ENGL acceptance criteria of 25% across the entire dynamic range of the assay [42].

The lowest concentration of analyte tested by the LGA as part of their validation was 0.1%. Therefore, according to their validation study, the LOQ is at or below 0.10%. As part of the formal validation, the absolute Limit of Quantitation was found to be 40 genomic copies or lower, which is consistent with the relative LOQ determined during method development.

In summary, the validation carried out by the Laboratory for GMO Analysis of the Umweltbundesamt GmbH established that the SU canola-specific qPCR method meets all criteria for GMO testing methods established by the ENGL and by the Bundesamt für Verbraucherschutz und Lebensmittelsicherheit *Guidelines for the single-laboratory validation of qualitative real-time PCR methods* [33,34,35,36,42].

## 4. Discussion

We present in this paper the first experimental evidence addressing the feasibility of developing GMO regulation-compliant analytical methods for identification and quantitation of genome-edited plant material.

The recent report by ENGL [28] as well as a number of other articles (for instance [7,10,25,29]) have raised doubts regarding whether it is possible to create accurate and sensitive PCR methods for detection and quantitation of genome-edited plant materials that meet requirements for testing methods set out in the GMO regulations of the EU and similar jurisdictions. Our work provides a definitive answer to this question, demonstrating that highly sensitive and specific PCR methods that meet GMO regulatory requirements can be developed to detect and quantify edited organisms even when the genome edit consists of only a single base pair alteration, and even in cases of multicopy gene targets. This can be achieved even in complex allotetraploid genomes such as that of canola.

Based on the success with this challenging case of genomic editing, we are optimistic that standard qPCR, augmented with strategies such as the use of LNAs in primer design, will prove capable of delivering quantitative detection methods that meet GMO regulatory standards for all classes of genome-edited organisms, including single-nucleotide edits, as well as the indels and larger inserts for which qPCR methods are well established and in use for quantitative detection of GMOs created using recombinant DNA methods. Factors such as genome size and the possibly constrained sequence context of a given SNV (challenges such as the SNV being imbedded in an AT-rich, repetitive or primer–dimer-forming region) can also influence the sensitivity and specificity of the tests that can be developed for a given genome-edited event. However, a number of strategies are available for optimizing performance of the primer-probe set for a given SNV. In the present case, it was incorporation of an LNA residue into the reverse primer that led to a significant increase in specificity of the *AHAS1C SU* primer-probe system. Other strategies may be successful in other situations. Adjusting primer position by just a few bases can increase specificity by as much as 7 or 8 Ct, and targeting GC-rich regions as primer sites, incorporating mis-matches into primer sequence and use of minor groove binding for probes are additional strategies that have yielded practical success.

Such methods are compatible with the basic standards of practice, equipment and molecular biological expertise found in most regulatory and commercial GMO laboratories, and could be readily integrated into the analytical routine and infrastructure of these laboratories. They also meet the current ENGL requirements [42] for GMO detection and quantitation methods.

Because of the complexity of the canola *AHAS* gene family, the method that we developed for specific detection of SU canola relied on the presence of two SNVs, one at the site of genome editing, and a second that distinguished the *AHAS1C* gene from the *AHAS3A* gene. However, in most cases, targeting a single SNV at a defined location in the genome of the genome-edited organism will be sufficient to achieve definitive detection and quantitation of the genome-edited event. This was not the case for SU canola because it carried an *AHAS3C* gene with the same G-to-T alteration (brought about by chemical mutagenesis is) that was effected by genome editing in the *AHAS1C* gene of SU canola. There may be other infrequent exceptions in which a combination of markers may be needed, depending on the specifics of the gene of interest and its genomic context. In each case, it will be necessary to identify gene and genomic features that are distinctive for the genome-edited event of interest and design the test around those features. It is ideal for a method to focus on one SNV, if possible, since this makes it possible to minimize amplicon length. In the case of SU canola, the amplicon length turned out to be 334 bases, which carries the disadvantage that, for samples in which the DNA has been partially degraded by food processing, sensitivity will be reduced as amplicon length increases [43].

In the case of deletion or insertion of nucleotides via genome editing techniques, developing definitive qPCR detection methods will be even more straightforward than for single base pair edits. For instance, in the case of the Calyxt high-oleic soy, the TALEN nuclease effected deletions of approximately 4 to 63 base pairs at defined positions within soy fatty acid desaturase genes. Because the length of deletions cannot be precisely controlled using the TALEN method, the exact sequence at the deletion site is randomly determined and therefore unique since the probability of generating, by any mechanism, an identical deletion of exactly the same length is extremely low. Thus, a primer that spans the deletion point will uniquely differentiate that event.

At present, the model that most laboratories employ in GMO surveillance is called the matrix approach, where a series of sequence features common to many GMOs, such as the CaMV P-35S promoter and the T-nos terminator are targeted, along with event-specific targets for GMOs that do not carry common sequences [44,45]. The result is a minimal set of PCR targets that, together, are capable of efficiently detecting a broad range of authorized GMOs and known-unauthorized GMOs. To incorporate genome-edited GMOs into this scheme is straightforward: the sequence target or targets for each genome-edited GMO are incorporated into the screening matrix along with target sequences for other GMOs that do not carry common sequences. The number of genetically modified crops lacking common sequences has been increasing in recent years, and genome-edited crops will add to this number. Thus, screening will require an increasing number of primer-probe sets in the future. This could become economically unsustainable, if the field continues to rely on real-time qPCR. However, the use of next-generation sequencing technology’s capacity to carry out massively parallel multiplexed quantitative amplicon sequencing provides a viable alternative, although it will require significant development effort. This approach has already been applied qualitatively in GMO screening [46]. Fields such as microbiological ecology have already applied this approach quantitatively [47].

Discussions regarding the ability to develop methods for detection and quantitation of genome-edited crops often focus on the challenge of definitively demonstrating whether a particular genome modification is the result of genome editing or, for instance, chemical mutagenesis. However, a close reading of Directive 2001/18/EC [5] and Commission Implementing Regulation (EU) No 503/2013 [31] indicates the capacity for such discrimination is not necessary to meet the requirements for marketplace surveillance of GMOs, established in European Union law:

“The method(s) shall be specific to the transformation event (hereafter referred to as ‘event-specific’) and thus shall only be functional with the genetically modified organism or genetically modified based product considered and shall not be functional if applied to other transformation events already authorized; otherwise the method cannot be applied for unequivocal detection/identification/quantification.”—Section 3.1.C.1 of Annex III to Regulation (EU) No 503/2013

This passage from the EU Commission’s implementing regulation clearly does not require that GMO analytical methods must be able to ascertain the process by which a particular GMO was created. It only requires the analytical method to be able to distinguish the modified event from other authorized events in the marketplace. Language similar to that quoted above from Regulation (EU) No 503/2013 is also found in the ENGL document, Definition of Minimum Performance Requirements for Analytical Methods of GMO Testing [42]. Based on these authoritative sources, it is clear that methods such as the one reported in this paper for SU canola meet the event specificity requirements for GMO analytical methods under EU regulations.

We based development of the detection and quantitation method for SU canola on information available in the public domain, referenced earlier in this paper. More broadly such information would include GMO-related patents, documents related to governmental regulatory approvals, communications to investors and market information relevant to commercialization. This information is accessible to any interested party so that it can be systematically monitored and used for development of test methods. It is unlikely that a commercial product could enter the global food system without sufficient information being released into the public domain to adequately inform the method development process. In certain jurisdictions, such as the EU, information related to detection methods is available through another route, as well; GMO regulatory law requires commercial entities, seeking market authorization for genetically modified food products, to provide a product-specific detection and quantitation method. They are also required to make available reference material. Since the European Court of Justice has already established that genome-edited products fall within the scope of the EU GMO regulation [4], methods for detection and quantitation of genome-edited products must be made available by the developer as part of the market authorization process for every genome-edited product.

A final issue raised in discussions regarding methods for detection and quantitation of genome-edited crops is challenges related to detection of unauthorized genome-edited crops. In the past, commodities have been screened for unauthorized GMOs using tests that detect common sequence elements, such as the CaMV P-35S promoter and the T-nos terminator that have been used in the construction of many GMOs. It has been argued that genome-edited products are challenging because they do not carry these common sequences and therefore unauthorized genome-edited crops could not be detected using these broad screening methods [28]. It should be pointed out that this is not a limitation exclusive to genome-edited products. As discussed at length in a report from ENGL [28] and elsewhere [44,45], it is not difficult, using even recombinant DNA methods, to develop GMOs that are free from common sequence elements. Consequently, many such GMOs have been commercialized, and it is quite possible that there are unapproved GMOs in the marketplace, even now, that have not been detected because they do not carry any common sequences. Thus, unauthorized genome-edited products represent just one new class of unauthorized genetically modified products, among others, that cannot be detected by using the existing screening strategy. Screening for unauthorized GMOs is not, and never has been, an exhaustive process, and the presence of genome-edited products in the commercial food system does not create a new set of circumstances that demands fundamental changes in the regulatory regime for GMOs.

Although our work demonstrates that it may be possible to develop event-specific, GMO regulation-compliant detection methods for virtually any gene-edited organism based on information disclosed by the developer or gathered from the public domain, screening methods capable of detecting whole classes of gene-edited products, such as CRISPR or TALEN, would also be a useful addition to the screening matrix. CRISPR and TALEN modified organisms are often transgenic due to the incorporation into the host genome of the genetic apparatus that synthesizes the sequence- specific nuclease involved. Demorest et al. [48], as an example, reported that 80% of the TALEN events that they generated were transgenic. Screening methods for CRISPR or TALEN could, for instance, rely on retained transgenic fragments unintentionally left in the final plants’ genomes. There are attempts to select events that do not carry these sequences [49] or to use strategies that do not require such expression vectors [50], but there is quite limited empirical evidence at this time demonstrating the success and generalizability of these efforts. We expect that research in coming years will lead to screening methods for at least some, if not all, categories of genome-edited products.

## 5. Conclusions

We have developed a sensitive, GMO regulation-compliant method for detecting the first genome-edited crop to be commercialized and suggest that it may represent a general approach for detecting genome-edited organisms. This effort was intended to address the concern voiced by several [7,10,25,26,28,29] that it may not be possible to develop quantitative detection methods for genome-edited plant materials that meet GMO regulatory requirements in jurisdictions such as the EU. In particular, they questioned the feasibility of developing quantitative methods that target single-nucleotide edits and that are sufficiently sensitive and specific to meet the requirements of EU law and regulations. The research presented here provides a clear answer to these concerns, showing that straightforward qPCR methods can be developed for single-nucleotide edits (SNVs). This, in conjunction with the fact that the scientific community has been using qPCR to quantitatively detect indels and inserted genes for two decades, indicates that it may be possible to develop qPCR methods for virtually any genome edit.

Our approach does not rely on specialized point mutation detection procedures, but employs straightforward real-time qPCR methods that are compatible with the basic standards of practice, equipment and molecular biological expertise found in most regulatory and commercial GMO testing laboratories. The resulting methods can readily integrate into the analytical routine of the typical regulatory or commercial GMO testing laboratory, including the matrix approach. The SU canola assay has been independently validated and fully meets the requirements for GMO testing methods laid down in European Union law and regulations and has been placed in the public domain, accessible to all laboratories.

This work establishes the basis for developing a test-based strategy for monitoring genome-edited plant products that integrates seamlessly into the same strategy that is used today in the EU and most other countries to monitor and regulate GMOs developed through recombinant DNA methods. Such an approach will deliver the transparency that consumers are increasingly demanding for the food that they provide to their families, and will, if necessary, provide the post-market traceability needed in case of unintended biosafety, environmental or socio-economic impacts.

## Figures and Tables

**Table 1:** Sequences of primers and probes used. +A denotes the locked nucleic acid (LNA) version of A; FAM, 6-carboxyfluorescein; -BHQ, Black Hole Quencher-1.

Name	Primer Sequence (5′ to 3′)	Position	Amplicon Length	Reference
	**SU Canola-Specific Primers and Probe**			
SU-Forward Primer	TCC TCG ACG AGC TAA CCG	1364–1381	334	This Study
SU-Reverse Primer	GCT TTG TAG AAC CGA TCT TCC +A	1676–1697		
SU-Probe	FAM-ACA ACC AGC ATC TTG GGA TGG TCA-BHQ	1646–1669		
	**Endogenous Reference Primers and Probe**			
*CruA*-Forward Primer	GGC CAG GGT TTC CGT GAT	1408–1425	101	[39]
*CruA*-Reverse Primer	CCG TCG TTG TAG AAC CAT TGG	1488–1508		
*CruA*-Probe	FAM- AGT CCT TAT GTG CTC CAC TTT CTG GTG CA-BHQ	1427–1455		

**Table 2:** Real-time qPCR amplification of SU canola and Clearfield canola DNA with PCR systems specific for the canola endogenous reference gene *CruA* and for the *AHAS1C-SU* gene of SU canola. DNA isolated from three SU canola varieties, C1511, C5507, and 40K, and three Clearfield canola varieties, 5545 CL, CS220 CL, and 2022 CL, were tested with primers specific for the *AHAS1C-SU* gene or for the *CruA* gene, with 200 ng input DNA per reaction. PCR conditions are as described in Methods. For all conditions except water control, *n* = 3. ND indicates that no amplification was detected; NA indicates “Not Applicable” because no amplification was observed.

PCR Specificity	*AHAS1C-SU*		*CruA*	
Canola Variety	Mean Ct	Ct% CV	Mean Ct	Ct% CV
5545 CL	ND	NA	20.81	0.16%
CS2200 CL	ND	NA	21.24	0.48%
2022 CL	ND	NA	21.02	0.16%
C5507	23.71	0.15%	20.93	0.09%
C1511	23.40	0.08%	20.87	0.10%
40K	22.37	0.41%	20.55	0.29%
Water	ND	NA	ND	NA

**Table 3:** Real-time qPCR amplification of DNA from SU canola event 40K, Clearfield canola variety 5545 CL and 20 varieties of wild-type canola with PCR systems specific for the *CruA* endogenous canola reference gene and the *AHAS1C-SU* SNV present in SU canola. PCR conditions are as described in Methods, with 300 ng input DNA per reaction for each variety except that the concentrations of DNA in the reactions labeled 40K-10, 40K-1 and 40K-0.1 were 300, 30 and 3 ng DNA of 40K DNA, respectively, with total DNA adjusted to 300 ng with DNA from canola variety 5454 CL for reactions 40K-1 and 40K-01. For all wild-type varieties, *n* = 4. ND indicates that no amplification was detected; NA indicates “Not Applicable” because no amplification was observed.

PCR Specificity	*AHAS1C-SU*		*CruA*	
Canola Variety	Mean Ct	Ct% CV	Mean Ct	Ct% CV
40K-10	25.36	0.08%	19.88	0.20%
40K-1	28.19	2.09%	20.10	0.72%
40K-0.1	33.43	1.44%	20.22	0.12%
5545 CL	ND	NA	20.88	0.46%
Variety 1	ND	NA	21.25	0.20%
Variety 2	ND	NA	19.64	0.75%
Variety 3	ND	NA	20.15	0.35%
Variety 4	ND	NA	19.45	0.11%
Variety 5	ND	NA	19.37	0.52%
Variety 6	ND	NA	18.74	0.49%
Variety 7	ND	NA	19.27	0.19%
Variety 8	ND	NA	19.19	0.48%
Variety 9	ND	NA	19.22	0.55%
Variety 10	ND	NA	19.67	0.73%
Variety 11	ND	NA	19.56	0.78%
Variety 12	ND	NA	19.12	0.38%
Variety 13	ND	NA	19.45	0.19%
Variety 14	ND	NA	19.08	0.49%
Variety 15	ND	NA	19.62	0.28%
Variety 16	ND	NA	20.47	1.12%
Variety 17	ND	NA	21.23	0.20%
Variety 18	ND	NA	19.06	0.50%
Variety 19	ND	NA	23.65	0.26%
Variety 20	ND	NA	18.29	0.39%
No DNA	ND	NA	34.36	2.48%

**Table 4:** Real-time quantitative PCR analysis of SU canola DNA, variety 40K, mixed at five concentrations with Clearfield canola variety 5545 CL. Total DNA was 300 ng/reaction and *n* = 12. PCR conditions, as described in Methods using the SU canola-specific primers and probe. % CV indicates % Coefficient of Variation.

Declared SU Canola DNA Conc.	Average Measured SU Canola DNA Conc.	Std Dev of DNA Conc.	% CV of DNA Conc.	Percent Trueness of Measured DNA Conc.	Average Ct	Std Dev of Ct	% CV of Ct
10.00%	8.631%	0.758	8.8	13.69%	25.6490	0.1545	0.60
1.00%	1.011%	0.050	5.0	1.10%	29.0010	0.0762	0.26
0.10%	0.103%	0.012	11.1	3.38%	32.5780	0.1798	0.55
0.05%	0.045%	0.006	16.8	6.93%	33.8381	0.2587	0.76
0.01%	0.004%	0.003	69.0	56.68%	37.8368	0.9926	2.62

## Supplementary Materials

The following are available online at https://www.mdpi.com/2304-8158/9/9/1245/s1, Table S1 List of Wild Type Canola Varieties, Table S2 Complete sequences from Sanger sequencing of four canola varieties, Table S3 PCR amplification of SU canola and Clearfield canola DNA with *CruA* and SU canola specific primer sets, Table S4. Quantitative PCR amplification of DNA from SU canola, event 40K, Clearfield canola variety 5545 CL and 20 varieties of wild type canola with *CruA* and SU canola-specific primer sets, Table S5a,b Determination of Limit of Quantitation, Limit of Detection, Trueness and Precision for Quantitative PCR System Specific for *AHAS1C SU* and S6 AEA Validation Report, File S1—Statement Regarding Associated Intellectual Property.
