# Correction: Chhalliyil et al. A Real-Time Quantitative PCR Method Specific for Detection and Quantification of the First Commercialized Genome-Edited Plant. *Foods* 2020, *9,* 1245

**DOI:** 10.3390/foods11040585

**Published:** 2022-02-18

**Authors:** Pradheep Chhalliyil, Heini Ilves, Sergei A. Kazakov, Stephanie J. Howard, Brian H. Johnston, John Fagan

**Affiliations:** 1 Health Research Institute, 505 Dimick Drive, P.O. Box 370, Fairfield, IA 52556, USA; pradheep@hrilabs.org; 2 Somagenics, Inc., 2161 Delaware Ave, Suite E, Santa Cruz, CA 95060, USA; hilves@somagenics.com (H.I.); skazakov@somagenics.com (S.A.K.); bjohnston@somagenics.com (B.H.J.); 3 The Sustainability Council of New Zealand, P.O. Box 24304, Wellington 6142, New Zealand; stephanie.howard@sustainabilitynz.org

## Text Correction

The stated objective of the paper was to provide regulatory laboratories and industry laboratories a complete, legally robust method for the quantitative detection of SU canola. Feedback from scientists operating in regulatory laboratories clearly signaled that a distinct set of systematic criteria by which the scientist operating the method could consistently judge results to indicate the presence or absence of SU canola, and, if present, to provide accurate quantitation thereof are essential to deliver the stated objectives of the paper. Such criteria are of particular importance in cases of the possible low-level presence of SU canola. The purpose of the correction is to rectify this omission. The authors apologize for any inconvenience caused and state that the scientific conclusions are unaffected. The authors make the following corrections to their published paper [1]. The original publication has also been updated.

Section 3.2.* Specificity of the SU Canola-Specific qPCR Method* has been corrected. For clarity, the full text of Section 3.2 is inserted below (without figures and tables) with changes. The corrections are as follows: (1) in Section 3.2, paragraph 2 small edits in bold have been made to lines 8, 9 and 12. (2) Three paragraphs have been added at the end of the section. 

### 3.2. Specificity of the SU Canola-Specific qPCR Method

We evaluated the specificity of these primer sets using DNA isolated from Clearfield canola varieties that carry the G-to-T mutation at position 1667 in the *AHAS3A* gene, but not in the *AHAS1C* gene and from SU canola DNA, which carries the G-to-T mutation at position 1667 in the *AHAS3A* gene and at position 1676 in the *AHAS1C* gene. As shown in Table 2 and Figure 4, while the *CruA* PCR system amplified both Clearfield and SU canola DNA, the SU canola-specific PCR system amplified only the DNA from SU canola. There was no amplification of the water/no-template control. As expected, since C5507 and C1511 appear to be heterozygous based on Sanger sequencing, while 40K appears to be homozygous (see Figure 2), the Ct for 40K with the SU canola primer-probe set was roughly 1 Ct lower than that of C1511 and C5507. See Table S3 Supplementary Materials for original data.

To confirm the specificities of the *AHAS1C-SU* PCR system for SU canola, we tested its ability to amplify *AHAS1C* sequences from 20 different wild-type canola varieties. Whereas the primer set targeting the *CruA* canola reference gene amplified DNA from all 20 wild-type varieties as well as DNA from the three SU canola varieties and a Clearfield canola variety (Figure 4A), the SU canola-specific primer set failed to amplify DNA from any of the 20 varieties of wild-type canola or from the Clearfield variety but did amplify all three of the SU canola DNA positive controls (Figure 4B). Weak background amplification was observed in a few reactions of **two** of the wild-type varieties, but this was not consistent among replicates. Of the **93** replicates run with wild-type or Clearfield canola, only **3** showed amplification, and this occurred only at very high cycle numbers, 41 or greater, in contrast to a Ct of 25.3 for an equivalent concentration of SU canola DNA. See **Table S3 (Clearfield) and Table S4 (wild-type)** in Supplementary Materials for original data, which shows lack of consistency among replicates. Weak background was also seen for the no DNA control for the *CruA* but not the *AHAS1c* SU primer set. For *CruA*, the difference in Ct between the no DNA sample and samples containing 300 ng DNA was 14.9, which clearly differentiates background from real signals.

The 20 wild-type varieties tested are representative of canola varieties in production globally, including varieties from Bangladesh, Canada, China, Denmark, France, Germany, Japan, The Netherlands, New Zealand, Poland, Russia, South Korea, Sweden, and the United States. See Supplementary Materials Table S1 for accession numbers and the origins of all varieties used. From the results presented in Tables 2 and 3 and Figures 4 and 5, we conclude that the SU canola-specific qPCR method is highly selective for SU canola and does not detect the wild-type or Clearfield canola varieties tested.

Based on the above work, when low level presence of GM material can be expected, the following protocol provides a consistent and standardized approach for routine surveillance testing, consistent with regulatory norms of the EU and other jurisdictions, for determining whether SU canola has been detected with the *AHAS1C-SU* PCR system.

First, as specified [2,3], in most cases the amplification threshold is set at 10-times the standard deviation of the baseline fluorescence. To establish the baseline fluorescence value and determine the standard deviation, the baseline fluorescence is sampled from Ct = 3 to two Cts preceding the Ct of the most abundant sample in the run [2,3]. This corresponds to the default or auto setting for the threshold in most PCR systems. Second, amplification profiles with Ct values less than 32 are categorized as specific detection of SU canola. Third, amplification profiles with Ct values of 38 or greater are categorized as non-specific amplification. In this case, amplifications observed are considered background noise from quantitative real-time PCR chemistry. Fourth, amplification profiles with Ct values ranging from 32 up to 38 shall be categorized as inconclusive and require confirmation before being declared positive or negative for the presence of SU canola. As is apparent from Figure 6, a Ct of 32 corresponds roughly to the limit of detection of the *AHAS1C-SU* PCR system, established by the LGA (data discussed below and presented in detail in Supplementary Materials, Item 6). Fifth, when such an amplification profile is observed, the sample should be rerun in 12 replicates. Only if all 12 replicates are positive should the result be considered a candidate positive. These criteria are consistent with the specifications for determining the limit of detection set out in the ENGL’s Definition of Minimum Performance Requirements for Analytical Methods of GMO Testing [4] (i.e., the reference #40 in the original paper).

For final confirmation, in cases where all 12 replicates yield amplification profiles, the amplicons from two of the positive replicates of the candidate positive sample should be subjected to Sanger sequencing, essentially as described in Section 2.2 of Methods, except using the *AHAS1C-SU* PCR primers. Only if the sequence of the amplicon is confirmed to match that of *AHAS1C-SU* would the sample be declared as positive for SU canola. This sequence confirmation is necessary to establish a legally defensible basis for declaring such weak PCR amplifications as definitively indicating the presence of SU canola. In cases where the Sanger sequencing does not match the *AHAS1C-SU* sequence, the sequence data can be used to design a qPCR test, which can be used in the context of the matrix approach, to routinely distinguish that canola variant from SU canola.
