# Supplementary material for: A Real-Time Quantitative PCR Method Specific for Detection and Quantification of the First Commercialized Genome-Edited Plant
Source: Foods. 2020 Sep 7;9(9):1245. doi: 10.3390/foods9091245 (PMC7556030; doi:10.3390/foods9091245)
Supplement: Supplementary file 1 [file foods-09-01245-s001.zip › Supplementary Materials/S1-S4 Supplementary Materials-Tables S1 - S4.pdf]

Supplementary Materials

**Table S1 List of Wild Type Canola Varieties**

| Country of Origin | Variety Name | Accession Number |
|---|---|---|
| Bangladesh | BAU-M/50 | PI 432392 |
| Canada | BO-63 | Ames 15651 |
| Canada, Ontario | Jet Neuf | PI 649127 |
| Canada, Saskatchewan | Golden | PI 649126 |
| China | Gan You no. 4 | PI 436557 |
| Denmark, Fyn | Viking | PI 601266 |
| France | Tantal | PI 383422 |
| France | France 11 | PI 469799 |
| Germany | Laura | PI 458952 |
| Japan | Norin 3 | PI 469954 |
| Korea, South | Aomori-1 | PI 469726 |
| Netherlands | Arco C10-2 | Ames 15650 |
| New Zealand | 77-58 | PI 458965 |
| Poland | Poland 5 | PI 470007 |
| Poland, Wroclaw | Ames 2778 | Ames 2778 |
| Soviet Union, Former | Russia 6 | PI 470022 |
| Sweden | Comet | PI 649130 |
| Sweden, Malmohus | Topas | PI 601201 |
| United States, California | Siberian | Ames 26626 |
| United States, Oregon | 77-70 | PI 458979 |

**Table S2** PCR amplification of SU canola and Clearfield canola DNA with *CruA* and SU canola specific primer sets. DNA isolated from three SU canola events, C1511, C5507, and 40K, and three Clearfield canola varieties, 5545 CL, CS220 CL and 2022 CL were challenged with primers specific for SU canola or for the *CruA* gene, 200 ng DNA per reaction. PCR conditions as described in Methods. For all conditions except water control, n=3. ND indicates that no amplification was detected. NA indicates “not applicable”, because no amplification was observed.

| Sample ID | Ct | | Ave Ct | | Std Dev Ct | | Rel Std Dev Ct (%) | |
|---|---|---|---|---|---|---|---|---|
| | SU Canola Primers | CruA Primers | SU Canola Primers | CruA Primers | SU Canola Primers | CruA Primers | SU Canola Primers | CruA Primers |
| 5545 CL | | 20.7839 | ND | 20.8573 | NA | 0.0660 | NA | 0.32 |
| 5545 CL | | 20.8499 | | | | | | |
| 5545 CL | | 20.8060 | | | | | | |
| C5507 | 23.7372 | 20.9056 | 23.7098 | 21.0294 | 0.0345 | 0.1436 | 0.15 | 0.68 |
| C5507 | 23.7211 | 20.9412 | | | | | | |
| C5507 | 23.6710 | 20.9296 | | | | | | |
| CS2200 CL | | 21.2058 | ND | 21.1852 | NA | 0.0291 | NA | 0.14 |
| CS2200 CL | | 21.1646 | | | | | | |
| CS2200 CL | | 21.3600 | | | | | | |
| 2022 CL | | 21.0245 | ND | 20.9552 | NA | 0.0887 | NA | 0.42 |
| 2022 CL | | 20.9790 | | | | | | |
| 2022 CL | | 21.0462 | | | | | | |
| C1511 | 23.3823 | 20.8426 | 23.0039 | 20.7361 | 0.5469 | 0.1827 | 2.38 | 0.88 |
| C1511 | 23.4061 | 20.8837 | | | | | | |
| C1511 | 23.4181 | 20.8706 | | | | | | |
| 40K | 22.4625 | 20.4839 | 22.3650 | 20.5499 | 0.0912 | 0.0595 | 0.41 | 0.29 |
| 40K | 22.3506 | 20.5995 | | | | | | |
| 40K | 22.2818 | 20.5663 | | | | | | |
| water | | | | | | | | |
| water | | | | | | | | |

**Table S3.** Quantitative PCR amplification of DNA from SU canola, event 40K, Clearfield canola variety 5545 CL and 20 varieties of wild type canola with CruA and SU canola-specific primer sets. PCR conditions are as described in Methods, with 300 ng input DNA per reaction for each variety except that the concentration of 40K DNA in the reactions labeled 40K-10, 40K-1 and 40K-0.1 were 300, 30 and 3 ng DNA 40K DNA per reaction, respectively, with total DNA adjusted to 300 ng for these reactions with DNA from canola variety 5454 CL. The n = 4 for all wild-type varieties. ND indicates that no amplification was observed. N/A indicates "Not Applicable".

| Well | Canola Variety | Ct CruA Primer Set | Mean Ct CruA Primer Set | Std Dev Ct CruA Primer Set | Ct SU Canola Primer Set | Mean Ct SU Canola Primer Set | Std Dev Ct SU Canola Primer Set |
|---|---|---|---|---|---|---|---|
| A1 | 40K-10 | 19.876 | 19.904 | 0.04 | 25.356 | 25.343 | 0.02 |
| A2 | 40K-10 | 19.933 | | | 25.330 | | |
| A3 | 40K-1 | 20.099 | 20.202 | 0.15 | 28.191 | 28.608 | 0.59 |
| A4 | 40K-1 | 20.305 | | | 29.026 | | |
| A5 | 40K-0.1 | 20.221 | 20.239 | 0.02 | 33.433 | 33.094 | 0.48 |
| A6 | 40K-0.1 | 20.256 | | | 32.755 | | |
| A7 | No DNA | 34.360 | 34.963 | 0.85 | ND | ND | N/A |
| A8 | No DNA | 35.565 | | | ND | | |
| B1 | 5545 CL | 20.877 | 20.852 | 0.09 | ND | ND | N/A |
| B2 | 5546 CL | 20.822 | | | ND | | |
| B3 | 5547 CL | 20.741 | | | ND | | |
| B4 | 5548 CL | 20.966 | | | ND | | |
| B5 | Variety 1 | 21.254 | 21.193 | 0.04 | ND | ND | N/A |
| B6 | Variety 1 | 21.173 | | | ND | | |
| B7 | Variety 1 | 21.161 | | | ND | | |
| B8 | Variety 1 | 21.185 | | | ND | | |
| B9 | Variety 2 | 19.641 | 19.530 | 0.15 | ND | ND | N/A |
| B10 | Variety 2 | 19.627 | | | ND | | |
| B11 | Variety 2 | 19.531 | | | ND | | |
| B12 | Variety 2 | 19.321 | | | ND | | |
| C1 | Variety 3 | 20.154 | 20.231 | 0.07 | ND | ND | N/A |
| C2 | Variety 3 | 20.314 | | | ND | | |
| C3 | Variety 3 | 20.194 | | | ND | | |
| C4 | Variety 3 | 20.264 | | | ND | | |
| C5 | Variety 4 | 19.452 | 19.444 | 0.02 | ND | ND | N/A |
| C6 | Variety 4 | 19.429 | | | ND | | |
| C7 | Variety 4 | 19.424 | | | ND | | |

| | | | | | | | |
|---|---|---|---|---|---|---|---|
| C8 | Variety 4 | 19.472 | | | ND | | |
| C9 | Variety 5 | 19.371 | 19.306 | 0.10 | ND | ND | N/A |
| C10 | Variety 5 | 19.412 | | | ND | | |
| C11 | Variety 5 | 19.237 | | | ND | | |
| C12 | Variety 5 | 19.205 | | | ND | | |
| D1 | Variety 6 | 18.744 | 18.823 | 0.09 | ND | ND | N/A |
| D2 | Variety 6 | 18.745 | | | ND | | |
| D3 | Variety 6 | 18.892 | | | ND | | |
| D4 | Variety 6 | 18.911 | | | ND | | |
| D5 | Variety 7 | 19.273 | 19.242 | 0.04 | ND | ND | N/A |
| D6 | Variety 7 | 19.236 | | | ND | | |
| D7 | Variety 7 | 19.191 | | | ND | | |
| D8 | Variety 7 | 19.267 | | | ND | | |
| D9 | Variety 8 | 19.191 | 19.157 | 0.09 | ND | ND | N/A |
| D10 | Variety 8 | 19.254 | | | ND | | |
| D11 | Variety 8 | 19.147 | | | ND | | |
| D12 | Variety 8 | 19.034 | | | ND | | |
| E1 | Variety 9 | 19.217 | 19.358 | 0.11 | ND | ND | N/A |
| E2 | Variety 9 | 19.375 | | | ND | | |
| E3 | Variety 9 | 19.368 | | | ND | | |
| E4 | Variety 9 | 19.472 | | | ND | | |
| E5 | Variety 10 | 19.673 | 19.471 | 0.14 | ND | ND | N/A |
| E6 | Variety 10 | 19.390 | | | ND | | |
| E7 | Variety 10 | 19.349 | | | ND | | |
| E8 | Variety 10 | 19.472 | | | ND | | |
| E9 | Variety 11 | 19.561 | 19.459 | 0.15 | ND | ND | N/A |
| E10 | Variety 11 | 19.598 | | | ND | | |
| E11 | Variety 11 | 19.417 | | | ND | | |
| E12 | Variety 11 | 19.262 | | | ND | | |
| F1 | Variety 12 | 19.116 | 19.166 | 0.07 | ND | ND | N/A |
| F2 | Variety 12 | 19.096 | | | ND | | |
| F3 | Variety 12 | 19.203 | | | ND | | |
| F4 | Variety 12 | 19.250 | | | 41.542 | | |
| F5 | Variety 13 | 19.454 | 19.408 | 0.04 | ND | ND | N/A |
| F6 | Variety 13 | 19.405 | | | ND | | |
| F7 | Variety 13 | 19.407 | | | ND | | |
| F8 | Variety 13 | 19.367 | | | ND | | |
| F9 | Variety 14 | 19.078 | 19.051 | 0.09 | ND | ND | N/A |
| F10 | Variety 14 | 19.129 | | | ND | | |
| F11 | Variety 14 | 19.082 | | | ND | | |
| F12 | Variety 14 | 18.915 | | | ND | | |

| | | | | | | | |
|---|---|---|---|---|---|---|---|
| G1 | Variety 15 | 19.624 | 19.570 | 0.05 | ND | ND | N/A |
| G2 | Variety 15 | 19.501 | | | 43.841 | | |
| G3 | Variety 15 | 19.556 | | | ND | | |
| G4 | Variety 15 | 19.598 | | | 41.406 | | |
| G5 | Variety 16 | 20.468 | 20.211 | 0.23 | ND | ND | N/A |
| G6 | Variety 16 | 20.205 | | | ND | | |
| G7 | Variety 16 | 20.258 | | | ND | | |
| G8 | Variety 16 | 19.912 | | | ND | | |
| G9 | Variety 17 | 21.231 | 21.174 | 0.04 | ND | ND | N/A |
| G10 | Variety 17 | 21.180 | | | ND | | |
| G11 | Variety 17 | 21.150 | | | ND | | |
| G12 | Variety 17 | 21.134 | | | ND | | |
| H1 | Variety 18 | 19.064 | 18.990 | 0.10 | ND | ND | N/A |
| H2 | Variety 18 | 19.078 | | | ND | | |
| H3 | Variety 18 | 18.934 | | | ND | | |
| H4 | Variety 18 | 18.883 | | | ND | | |
| H5 | Variety 19 | 23.654 | 23.744 | 0.06 | ND | ND | N/A |
| H6 | Variety 19 | 23.753 | | | ND | | |
| H7 | Variety 19 | 23.796 | | | ND | | |
| H8 | Variety 19 | 23.772 | | | ND | | |
| H9 | Variety 20 | 18.286 | 18.224 | 0.07 | ND | ND | N/A |
| H10 | Variety 20 | 18.146 | | | ND | | |
| H11 | Variety 20 | 18.284 | | | ND | | |
| H12 | Variety 20 | 18.178 | | | ND | | |

**Table S4a.** Real time quantitative PCR analysis of SU canola DNA, variety 40K, mixed at 5 concentrations with Clearfield canola variety 5545 CL. Total DNA was 300 ng/reaction and n = 12. PCR conditions, as described in Methods using the SU canola-specific primers and probe.

| Declared Concentration | Log Conc of Replicates | DNA Conc of Replicates | Average DNA Conc | Std Dev of Average DNA Conc | Rel Std Deviation | Accuracy of Measured DNA Conc |
|---|---|---|---|---|---|---|
| % | | % SU Canola DNA in total DNA | % SU Canola DNA in total DNA | | % | % difference between measured conc and declared conc |
| 10 | 0.804591 | 6.3766 | 8.6311 | 0.7583 | 8.79 | 13.69 |
| | 0.927165 | 8.4560 | | | | |
| | 0.973636 | 9.4110 | | | | |
| | 0.951302 | 8.9393 | | | | |
| | 0.938817 | 8.6859 | | | | |
| | 0.946058 | 8.8320 | | | | |
| | 0.947224 | 8.8557 | | | | |
| | 0.951552 | 8.9444 | | | | |
| | 0.944200 | 8.7943 | | | | |
| | 0.961706 | 9.1560 | | | | |
| | 0.935072 | 8.6114 | | | | |
| | 0.929967 | 8.5107 | | | | |
| 1 | -0.023572 | 0.9472 | 1.0110 | 0.0503 | 4.98 | -1.10 |
| | -0.010532 | 0.9760 | | | | |
| | 0.007002 | 1.0163 | | | | |
| | -0.007064 | 0.9839 | | | | |
| | 0.008223 | 1.0191 | | | | |
| | 0.015408 | 1.0361 | | | | |
| | 0.025618 | 1.0608 | | | | |
| | 0.054278 | 1.1331 | | | | |
| | 0.004477 | 1.0104 | | | | |
| | -0.001183 | 0.9973 | | | | |
| | -0.000323 | 0.9993 | | | | |
| | -0.020937 | 0.9529 | | | | |
| 0.1 | -0.990040 | 0.1023 | 0.1034 | 0.0115 | 11.15 | -3.38 |
| | -1.009683 | 0.0978 | | | | |
| | -0.936466 | 0.1158 | | | | |

| | | | | | | |
|---|---|---|---|---|---|---|
| | -0.948757 | 0.1125 | | | | |
| | -1.076852 | 0.0838 | | | | |
| | -1.063867 | 0.0863 | | | | |
| | -1.034236 | 0.0924 | | | | |
| | -0.972478 | 0.1065 | | | | |
| | -0.947619 | 0.1128 | | | | |
| | -0.925202 | 0.1188 | | | | |
| | -0.996393 | 0.1008 | | | | |
| | -0.955859 | 0.1107 | | | | |
| 0.05 | -1.375018 | 0.0422 | 0.0465 | 0.0078 | 16.80 | 6.93 |
| | -1.395826 | 0.0402 | | | | |
| | -1.362700 | 0.0434 | | | | |
| | -1.251057 | 0.0561 | | | | |
| | -1.332320 | 0.0465 | | | | |
| | -1.206167 | 0.0622 | | | | |
| | -1.304603 | 0.0496 | | | | |
| | -1.469348 | 0.0339 | | | | |
| | -1.387447 | 0.0410 | | | | |
| | -1.351574 | 0.0445 | | | | |
| | -1.265928 | 0.0542 | | | | |
| | -1.350631 | 0.0446 | | | | |
| 0.01 | -2.527344 | 0.0030 | 0.0043 | 0.0030 | 69.01 | 56.68 |
| | -2.567878 | 0.0027 | | | | |
| | -2.263025 | 0.0055 | | | | |
| | -2.428935 | 0.0037 | | | | |
| | -2.798959 | 0.0016 | | | | |
| | -1.973487 | 0.0106 | | | | |
| | -2.211171 | 0.0061 | | | | |
| | -2.031167 | 0.0093 | | | | |
| | -2.811722 | 0.0015 | | | | |
| | -2.491970 | 0.0032 | | | | |
| | -2.643231 | 0.0023 | | | | |
| | -2.616819 | 0.0024 | | | | |

**S4b Definition of Limit of Quantitation and Calculation of Limit of Detection**

| Limit of Quantitation defined as the lowest concentration of analyte at which the Relative Standard Deviation is less than 25%. |
|---|
| LOQ = 0.050 % SU Canola DNA in 300 ng Total DNA |

| Calculation of Relative Limit of Detection |
|---|
| LOD = (SD)(t-value(pValue, n-1)<br>LOD = (0.008)(t-value(0.99. 12-1)<br>LOD = (0.008)(3.10580652) = 0.025 |

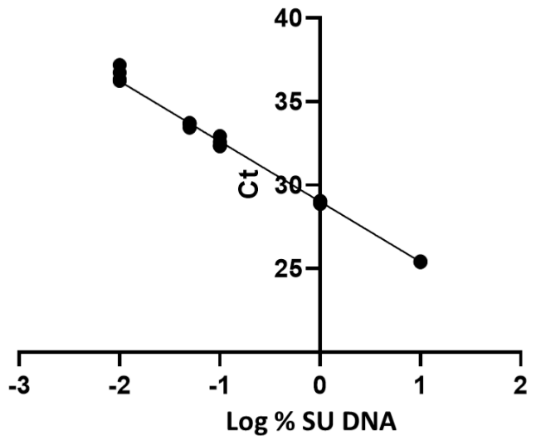

**S5 AEA Validation Report (Separate Document, PDF)**

See separate PDF
