# Supplementary material for: A Real-Time Quantitative PCR Method Specific for Detection and Quantification of the First Commercialized Genome-Edited Plant
Source: Foods. 2020 Sep 7;9(9):1245. doi: 10.3390/foods9091245 (PMC7556030; doi:10.3390/foods9091245)
Supplement: Supplementary file 1 [file foods-09-01245-s001.zip › Supplementary Materials/Statement Regarding Associated Intellectual Property - Chhalliyil et al.pdf]

File S1, Supplementary Materials

Statement Regarding Associated Intellectual Property

Consistent with the commitment to open source the detection method described in the article, "A Real-Time Quantitative PCR Method Specific for Detection and Quantification of the First Commercialized Genome-Edited Plant", we place on the public record the following statement with respect to the associated intellectual property.

1. Abstract

The disclosed method is a quantitative PCR (polymerase chain reaction) detection method for the first commercialized genome-edited crop, a canola with a single base pair edit conferring herbicide tolerance.

The method, validated by an independent laboratory, meets all legal requirements for GMO analytical methods in jurisdictions such as the EU and is consistent with ISO17025 accreditation standards.

This approach to qPCR-based method development, which we have used for the first commercialized genome edited crop, may also be applicable to the detection of virtually any genome-edited organism, for which at least minimal construct information is available.

2. Problem Statement

Doubts about the ability to detect genome-edited organisms have raised questions about whether current GMO regulations in many jurisdictions can operate effectively.

This is significant as the purpose of these regulatory regimes is to protect public health and the environment from risks that may arise from all GMOs, including those produced by new techniques such as gene editing.

Discussions regarding how detection and quantification of genome-edited organisms could be achieved have, to date, remained mostly theoretical.

If a detection method for the first commercialised genome-edited organism could be developed and open-sourced, this would provide an empirical basis for designing methods for subsequent new genetically modified organisms that could give confidence that existing regulatory regimes can continue to fulfil their purpose.

3. Known Solutions and Drawbacks

Although methods exist for single-nucleotide variant (SNV) genotyping and SNV allelic discrimination, these methods are not adaptable to routine analysis of commodity crops for the presence of GMOs, where the genetic modification consists of variations as small as a single base alteration.

Detecting such small variations requires a significant level of method optimization of SNV genotyping and SNV allelic discrimination methods, and the use of these methods requires a degree of technical experience that is not typically available in enforcement and commercial laboratories, dedicated to routine GMO testing of food and feed commodities.

These methods are also typically not sufficiently sensitive and discriminatory to meet current requirements for GMO detection methods when complex foodstuffs are analyzed.

What is needed are detection methods that are adaptable to existing equipment and expertise found in GMO laboratories, and that perform to a level consistent with the requirements for GMO analysis set out in the GMO regulations of jurisdictions such as the EU.

4. Novelty Statement

The method developed meets the need described above, enabling identification and quantification of a single nucleotide variant in a genome-edited organism.

The principal novel step is the use of optimization strategies to augment standard PCR approaches so that they are suitable for the detection and quantification of genome-edited GMOs. Those optimisation strategies include, but are not limited to, the following:

- Introducing locked nucleic acids (LNAs) into the primer design;
- Adjusting primer position by just a few bases, which can increase specificity by as much as 7 or 8 Ct;
- Targeting GC-rich regions as primer sites;
- Incorporating mis-matches into the primer sequence;
- Using minor groove binding for probes;
- Incorporating protein nucleic acids into primer design.

5. Implementation/Process Steps

The paper includes all information needed to implement the method. A scientist with experience in basic qPCR analytical methods will be fully capable of, stepwise, carrying out the process described in the manuscript. The preparation of all reagents is described; the application of those reagents in conjunction with a real time quantitative PCR instrument and other supplies and instrumentation are described. All of these can be applied systematically, as described in the paper, to accomplish the objective of high-sensitivity detection of a specific SNV.

6. Example Embodiments

Example 1: Border surveillance by regulatory authorities of canola imports: Regional blocks such as the European Union and countries such as New Zealand have zero tolerance policies to imports that contain genetically modified organisms (GMOs) that have not

received regulatory clearance to be placed on the market. The GM canola that this detection test has been developed for is one example of a GMO that has not received regulatory clearance in either the EU or New Zealand. The method allows regulators to uphold biosecurity laws, by physically testing canola import consignments for the presence of the GM canola and to respond appropriately.

Example 2: Food and feed companies use of the test to demonstrate compliance with non-GMO certification requirements: Achieving non-GM certification for food products provides significant market advantage for food and feed companies in many countries. Until now, companies working in supply chains that are at risk of contamination from this particular GM canola have had to rely on paper trial traceability to vouch for the integrity of their products. Now, food and feed companies can use the method – or require their suppliers to verify through use of it – to protect their supply chains.

Example 3: Environmental regulators use test to identify GM canola and take appropriate actions in the event that environmental assessments indicate that the canola is capable of actual or potential harmful effects on ecosystems or health.
