# Supplementary material for: A Real-Time Quantitative PCR Method Specific for Detection and Quantification of the First Commercialized Genome-Edited Plant
Source: Foods. 2020 Sep 7;9(9):1245. doi: 10.3390/foods9091245 (PMC7556030; doi:10.3390/foods9091245)
Supplement: Supplementary file 1 [file foods-09-01245-s001.zip › Supplementary Materials/S5 Supplementary Materials-Item 5 AEA Validation Report.pdf]

VALIDATION OF A METHOD TO DETECT AND QUANTIFY CIBUS® OILSEED RAPE USING REAL-TIME PCR

Validation Report

Frank Narendja
Laboratory for GMO Analysis

REPORT

Vienna, 21. December 2019

Content

1 STATEMENT 3

2 MATERIALS AND METHODS 4

2.1 Samples 4

2.2 DNA extraction 4

2.3 Method for the PCR analysis 4

2.4 Real-time PCR equipment used in the study 7

2.5 Materials used in the validation study 7

3 RESULTS 9

3.1 Method performance for qualitative PCR 9

3.1.1 Limit of Detection (LOD) 9

3.1.2 Probability of Detection (POD) 10

3.1.3 Specificity 10

3.1.4 Robustness 12

3.2 Method performance for quantitative PCR 14

3.2.1 PCR efficiency and linearity 14

3.2.2 Trueness and Precision 15

3.2.3 Limit of Quantification (LOQ) 15

4 REFERENCES 17

1 STATEMENT

A PCR based method for detection and quantification of the CIBUS gene edited oilseed rape event was developed by the method developer and submitted for validation to the Laboratory for GMO Analysis of the Umweltbundesamt GmbH (Environment Agency Austria). The Laboratory for GMO Analysis is accredited according to ISO 17025:2017.

Validation was conducted according to the “Guidelines for the single-laboratory validation of qualitative real-time PCR methods” of the German Federal Office of Consumer Protection and Food Safety [Bundesamt für Verbraucherschutz und Lebensmittelsicherheit (BVL)] as well as the ENGL guidance document “Verification of analytical methods for GMO testing when implementing interlaboratory validated methods - Version 2”. All PCR analyses were carried in line with the requirements of ISO24276, ISO21569 and ISO21570.

Positive and negative samples arrived on 06.09.2019. The protocols for DNA extraction and PCR conditions arrived on 03.11.2019. A modified protocol for PCR conditions were provided on 21.11.2019.

Experimental work was carried out from 25.11.2019 till 12.12.2019. Validation data and validation report were submitted on 21.12.2019.

Corrigendum (29.06.2020):

Page 4: size of CIBUS-specific PCR product was corrected

Page 11, table 9: footnotes were corrected

umweltbundesamt PERSPEKTIVEN FÜR UMWELT & GESELLSCHAFT	**Unterzeichner**	Frank Narendja
	**Datum/Zeit-UTC**	2020-06-29T15:32:00+02:00
**Prüfinformation**	Diese Unterschrift ist gemäß EU Verordnung Nr. 910/2014 (eIDAS) der handschriftlichen Unterschrift grundsätzlich rechtlich gleichgestellt. Prüfung unter: http://www.signaturpruefung.gv.at	

Vienna; 29.06.2010 Dr. Frank Narendja

(Head of Laboratory for GMO Analysis)

2 MATERIALS AND METHODS

2.1 Samples

The following positive and negative control samples were provided by the method developer as ground seeds Table 1:

Table 1: positive and negative control samples provided by the method developer

Sample code	event	trait
**CSP-421**	5545 CL	Clearfield
**CSP-422**	C5507	CIBUS
**CSP-423**	CS2200 CL	Clearfield
**CSP-424**	VT X121 CL	Clearfield
**CSP-425**	2022 CL	Clearfield
**CSP-426**	C1511	CIBUS
**CSP-428**	40K	CIBUS homozygous

Based on information from the method developer sample CSP-424 was omitted from validation study.

2.2 DNA extraction

Genomic DNA was isolated from ground oilseed rape material, using Qiagen DNeasy Plant Kit DNA extraction method with modifications according to the method developer. All DNAs used in this validation study were purified after extraction using GE Healthcare illustra™ MicroSpin™ S-300 spin column.

2.3 Method for the PCR analysis

The PCR method provided by the method developer is a real-time TaqMan® PCR procedure for detection and quantification of the relative content of CIBUS gene-edited oilseed rape DNA to total oilseed rape DNA. The procedure is a simplex system, in which a oilseed rape specific assay targeting the endogenous gene cruciferinA (cruA), and the CIBUS gene-edited oilseed rape target assay are performed in separate wells.

For specific detection of CIBUS gene-edited oilseed rape, a DNA fragment of 334-bp is amplified using specific primers. PCR products are measured during each cycle using target specific oligonucleotide primers

and a probe labelled with two fluorescent dyes: FAM (6-carboxyfluorescein) as reporter dye at the 5'-end and a BHQ-1 quencher dye at the 3'-end. The target sequence in the CIBUS gene-edited oilseed rape differs from Clearfield canola varieties and from wild-type canola varieties by a single basepair. The desired degree of selectivity was achieved by using LNA (Locked Nucleic Acid)-modified primers, see Table 2.

Table 2: primer and probes used in the validation study

primer	DNA Sequence (5' to 3')	Length (bases)
	**CIBUS Specific Gene**	
Cibus FWD	TCC TCG ACG AGC TAA CCG	18
Cibus-REV	GCT TTG TAG AAC CGA TCT TCC +A [1]	22
Cibus-Probe	5-FAM-ACAACCAGCATCTTGGGATGGTCA-BHQ	24
	**Endogenous Reference Gene (cruA)**	
Endo-FWD	ggC CAg ggT TTC CgT gAT	18
Endo-Rev	CCg TCg TTg TAg AAC CAT Tgg	21
Endo-Probe	AgT CCT TAT gTg CTC CAC TTT CTg gTg CA-TAMRA [2]	29

[1] +A Locked Nucleic Acid (LNA)
[2] In this validation study TAMRA was used instead of BHQ as quencher

PCR reaction conditions and reaction mixtures have been optimized by the method developer. Reaction mixtures were prepared according to Table 3 using the KAPA PROBE FORCE qPCR Master Mix (Biosystems/Roche). Additionally, for robustness test QuantiTect Multiplex qPCR Kit (Qiagen) was used.

Table 3: PCR reaction mixtures

CIBUS system	Stock	Final	Volume (ul)
**Forward Primer**	100uM	1600 nM	0.4
**Reverse Primer**	100uM	1600 nM	0.4
**Probe**	100uM	800 nM	0.2
**PCR Master Mix**			12.5
**DNA**		up to 300ng	5.0
**Deionized Water**			6.5
**Total**			25 ul

cruA system	Stock	Final	Volume (ul)
**Forward Primer**	100uM	800 nM	0.2
**Reverse Primer**	100uM	800 nM	0.2
**Probe**	100uM	400 nM	0.1
**PCR Master Mix**			12.5
**DNA**		up to 300ng	5.0
**Deionized Water**			7.0
**Total**			25 ul

For the relative quantification of CIBUS gene-edited oilseed rape, the oilseed rape taxon-specific system amplifies a 101 bp fragment of the oilseed rape cruciferinA (cruA) endogenous gene, using cruA gene-specific primers and a cruA gene-specific probe. The cruA gene probe used in this validation study was labelled with FAM (6-carboxyfluorescein) as reporter dye at its 5' end and TAMRA (6-carboxytetramethylrhodamine) as quencher dye at its 3' end.

Standard curves were generated for both the CIBUS gene-edited oilseed rape and the cruA systems by plotting the Cq values measured for the calibration points against the logarithm of the DNA amount and by fitting a regression line into these data. Thereafter, the standard curves were used to estimate the DNA amount in the test sample by interpolation from the standard curves. The values used in the quantification, the GMO contents of the calibration samples, and the total DNA quantity used in the PCR reactions are listed in Table 4 and 5. For quantification runs D and E the concentration of calibration standards were adapted to better fit the limit of quantification.

Table 4: Values for DNA amount of the standard curve samples used in run B and C

calibrator	S1	S2	S3	S4	S5
**Total amount of oilseed rape DNA in the reaction (ng)**	125	12,5	1,25	0,125	0,062
**amount of CIBUS gene-edited oilseed rape DNA in the reaction (ng)**	125	12,5	1,25	0,125	0,062

Table 5: Values for DNA amount of the standard curve samples used in run D and E

calibrator	S1	S2	S3	S4	S5
**Total amount of oilseed rape DNA in the reaction (ng)**	250	25	2,5	0,25	0,125
**amount of CIBUS gene-edited oilseed rape DNA in the reaction (ng)**	250	25	2,5	0,25	0,125

The absolute copy numbers for LOD/POD determination are calculated by dividing the sample DNA mass (nanograms) by the published average 1C value for the oilseed rape genome (1.15 pg).

2.4 Real-time PCR equipment used in the study

All tests were conducted on a ABI 7500 PCR machine. Additionally, for robustness test a BioRad CFX96 was used.

PCR amplification reactions were carried out using the Thermal Cycler Profile shown in Table 6.

Table 6: Thermal Cycler Profile

Stage	Temp.	Time	NoCycles
**Activation/Initial Denaturation**	95°C	600”	1
**Denaturation**	95°C	15”	45
**Annealing & Extension**	60°C	60”	

2.5 Materials used in the validation study

For the validation of the CIBUS gene-edited oilseed rape -specific method, test samples were provided by the method developer (see Table 1) as well as from the validating laboratory.

The provided samples were used by the Environment Agency Austria to prepare calibration standards and test samples (of known GMO content) by mixing homozygous CIBUS oilseed rape DNA (sample CSP-428) and non-GM oilseed rape DNA according to Annex 3 of the ENGL guidance document “Verification of analytical methods for GMO testing when

implementing interlaboratory validated methods - Version 2". The two DNA replicates for each GM-level tested were prepared from two independent extractions of sample CSP-428.

Additionally two other CIBUS varieties (CSP-422 and CSP-426) were tested to demonstrate specificity.

Samples of non-target DNA to test for specificity were from various conventional (non-GM) plant material as well as various GM oilseed rape varieties, see Table 7.

Table 7: non-target DNA tested

non-GM oilseed rape	GM oilseed rape	conventional crops
Libraska	GT73	soy
Honk	MON88302	maize
Liberato	Liberator L62	rice
Lirajet	T45	cotton
Artus	MS8	sugarbeet
Naida	RF3	potato
Zeus		
DK Expression		
Clearfield CSP-421		
Clearfield CSP-423		
Clearfield CSP-425		

3 RESULTS

3.1 Method performance for qualitative PCR

The method performance characteristics for qualitative PCR were validated according to the “Guidelines for the single-laboratory validation of qualitative real-time PCR methods” of the German Federal Office of Consumer Protection and Food Safety [Bundesamt für Verbraucherschutz und Lebensmittelsicherheit (BVL)].

3.1.1 Limit of Detection (LOD)

The limit of detection has been determined by a dilution series of five levels of target DNA (CSP-428), using a uniform concentration of non-target DNA (salmon sperm DNA 20ng/µl) for each dilution level. Possible inhibitory effects of the salmon sperm DNA has been checked, no inhibition was observed.

For each dilution level, 12 PCR replicate measurements were performed. The lowest dilution level (i.e .the lowest number of copies) for which all 12 replicates are positive is considered to be an approximate value for $LOD_{95\%}$, Table 8.

Table 8: result of the LOD determination

Nominal copy number of target sequence	no. of replicates	no. of positive
**40**	12	12
**20**	12	12
**10**	12	12
**5**	12	11
**3**	12	10
**1**	12	8

According to the definition in the above mentioned guideline the absolute Limit of Detection (LOD_{abs}) for the CIBUS gene edited-specific PCR method has been determined to be approx. 10 genomic copies of the target sequence.

3.1.2 Probability of Detection (POD)

Additionally to the procedure described under 3.1.1 an alternative approach was used to determine the sensitivity of this detection method. On basis of modelling a POD curve the LOD_{abs} at the 95 % confidence interval (Uhlig et al. (2014) were computed (online tool available under https://quodata.de/content/validation-qualitative-pcr-methods-single-laboratory), Figure 1.

Figure 1: POD curve and $LOD_{95\%}$

The $LOD_{95\%}$ is 4.446 with a 95 % confidence interval of [2.822, 7.001].The figure below summarises the results. The blue diamonds characterise the laboratory-specific RODs. The blue curve denotes the mean POD curve along with the corresponding 95 % confidence range highlighted as the grey band. The POD curve under ideal conditions is displayed as the black dashed curve.

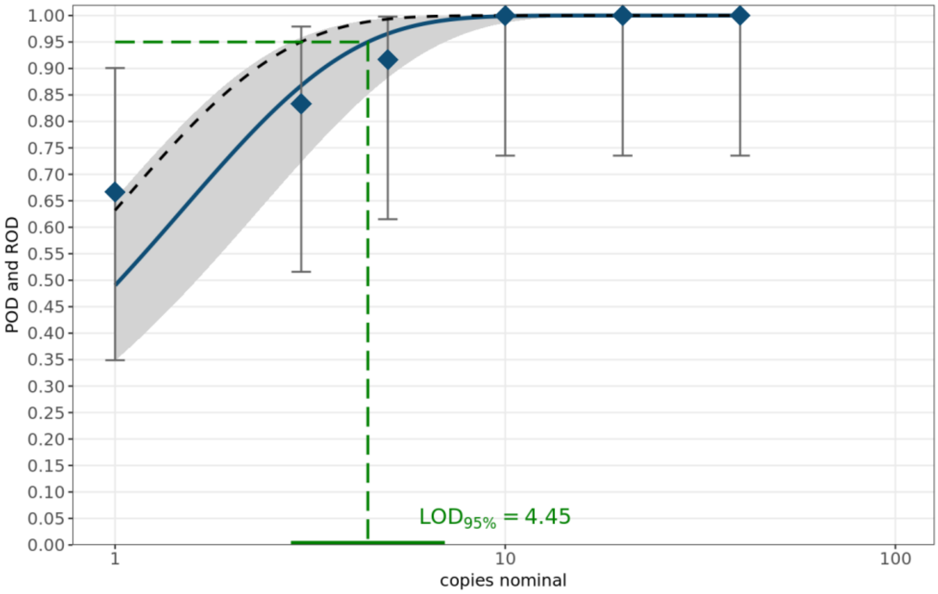

The result of the POD modelling indicates a value of 4,45 genomic copies for the $LOD_{95\%}$ which is in line with the LOD estimation above indicating that the $LOD_{95\%}$ of the method is between 5 and 10 genomic copies. Therefore, the LOD is in line with the ENGL acceptance criteria (< 25 copies with a level of confidence of 95%).

3.1.3 Specificity

In silico specificity analysis was carried out by the method developer.

The practical test for unexpected cross-reactions with non-target DNA was performed with a range of non-GM oilseed rape varieties as well as various GM oilseed rape events. In addition two other CIBUS varieties (CSP-422 and CSP-426) were tested to prove specificity. PCR reactions for each sample were performed in duplicates. Results are summarized in table 9.

All non target DNAs tested and expected to give a negative result were tested in concentrations of 50-100 ng/PCR reaction (≥ 2500 copies according to the guideline).

Target DNAs were tested at a concentration around LOQ according to the recommendations of the validation guideline.

Table 9: mean Cq-values for the CIBUS-specific PCR system and the cruA – specific PCR system

DNA tested		Cq-value CIBUS-specific system	Cq-value cruA-specific system
non-GM oilseed rape	Libraska	n.d.	24,7
	Honk	n.d.	25,1
	Liberato	n.d.	26,1
	Lirajet	n.d.	24,5
	Artus	n.d.	23,8
	Naida	n.d.	23,3
	Zeus	n.d.	24,1
	DK Expression	n.d.	24,6
	Clearfield CSP-421	n.d.	24,3
	Clearfield CSP-423	n.d.	23,7
	Clearfield CSP-425	n.d.	24,1
GM oilseed rape	GT73	n.d.	26,3
	MON88302	n.d.	25,2
	Liberator L62	n.d.	23,2
	T45	n.d.	21,4
	MS8	n.d.	21,5
	RF3	n.d.	21,4
CIBUS oilseed rape	CSP-422	36,2[1]	31,8[2]
	CSP-426	34,8[1]	31,0[2]

n.d. = not detected
[1] approx. 50 genomic copies (close to LOQ according to validation guideline)
[2] approx. 800 genomic copies

Additionally plant species which are often present in samples (corn, soya, rice, potato, cotton) were checked. None of the tested plants gave a positive signal with the CIBUS gene edited-specific PCR Method.

3.1.4 Robustness

A multifactorial experimental design was used to test for robustness of the PCR method. For every combination of the factor levels listed below, target DNA is added in a concentration around the LOQ (e.g.50 genomic copies). For each factor-level combination PCR tests were performed in triplicate.

To check the robustness of the real-time PCR method, the following conditions were tested:

- Real-time PCR equipment (2 different types or manufacturers)
- PCR reagent kit (2 different manufacturers)
- Annealing temperature (e.g. ±1 °C)
- Master mix volume (±5 %, e.g. 19 µl of master mix + 5 µl of DNA versus 21 µl of master mix + 5 µl DNA)
- Primer concentration (minus 30 %)
- Probe concentration (minus 30 %)

Tests were conducted on ABI 7500 and BioRad CFX96. The two different brands of PCR reagent kits were the KAPA PROBE FORCE qPCR Master Mix (Biosystems/Roche) and the QuantiTect Multiplex qPCR Kit (Qiagen).

Test was performed with 8 factor-level combinations as outlined in Figure 2.

Figure 2: factor-level combination design applied for robustness test

	factor-level combination							
	1	2	3	4	5	6	7	8
**PCR equipment**	ABI7500	ABI7500	ABI7500	ABI7500	CFX96	CFX96	CFX96	CFX96
**PCR reagent**	KAPA	KAPA	Qiagen	Qiagen	KAPA	KAPA	Qiagen	Qiagen
**Primer**	unchanged	-30%	unchanged	-30%	unchanged	-30%	unchanged	-30%
**Probe**	unchanged	-30%	-30%	unchanged	-30%	unchanged	unchanged	-30%
**Vol.**	19	19	21	21	21	21	19	19
**Temp.**	+1°C	-1°C	+1°C	-1°C	-1°C	+1°C	-1°C	+1°C

All factor-level combination except no. 4 gave positive results for all PCR replicates. With factor-level combination no. 4 one of the three PCR replicates was negative. However, according to the validation guideline this factor-level combination has been repeated and provided positive results for all PCR replicates in the second run. Based on the results of this test the CIBUS gene edited-specific PCR Method can be considered sufficiently robust.

3.2 Method performance for quantitative PCR

Samples with GM levels from 0.10 % to 5 % were tested in four real-time PCR runs (run B-E) with two replicates for each GM-level on each plate (total of eight replicates per GM-level) following the validation scheme "example 1" of the ENGL guidance document "Verification of analytical methods for GMO testing when implementing interlaboratory validated methods - Version 2". Data from run A were used for PCR efficiency and linearity only.

All quantitative tests were conducted on ABI 7500.

3.2.1 PCR efficiency and linearity

According to the ENGL method acceptance criteria, the average value of the slope of the standard curve shall range from -3.1 to -3.6 and the R^2 coefficient shall be ≥ 0.98. Results of five calibration runs are shown in Table 10.

Table 10. Standard curve parameters for slope and R2 coefficient of the real-time PCR tests, carried out on ABI 7500.

	CIBUS PCR system			cruA PCR system		
	Slope	PCR efficiency*	R^2	Slope	PCR efficiency*	R^2
**Run A**	-3.434	95,50	0,992	-3.283	101,62	0,998
**Run B**	-3.473	94,06	0,997	-3.409	96,47	0,997
**Run C**	-3.583	90,14	0,996	-3,385	97,45	0,999
**Run D**	-3,519	92,39	0,999	-3.461	94,49	0,997
**Run E**	-3,490	93,43	0,997	-3.271	102,18	0,999
**mean**	-3,500	93,10	0,996	-3,362	98,44	0,998

* PCR efficiency (%) is calculated using the formula Efficiency = $(10^{(-1/slope)}) - 1) \times 100$

Table 10 indicates that the slope and R^2 coefficient of the standard curves for the CIBUS gene edited-specific system and the oilseed rape-specific cruA system, as established by the method developer, were within the ENGL acceptance criteria.

3.2.2 Trueness and Precision

Calculations of means and variances were carried out according to the procedure outlined for random variables in the Annex 4 of the ENGL guidance document "Verification of analytical methods for GMO testing when implementing interlaboratory validated methods - Version 2". Results are shown in Table 11.

Table 11: Values of trueness and precision. GM % in m/m fraction

target %GM level	measured %GM level	Bias % of target GM level	Precision (RSDr %)
**5,00**	5,25	5,10	13,54
**1,00**	0,99	-0.12	10,29
**0,10**	0,11	14,76	16,19

Both parameters trueness and precision were within the ENGL acceptance criteria (trueness ± 25 %, RSDr ≤ 25 % across the entire dynamic range).

3.2.3 Limit of Quantification (LOQ)

According to the method developer, the relative LOQ of the method is at least 0.05 % in 200 ng of total oilseed rape DNA. The lowest relative GM content of the target sequence included in this validation study was 0.10 % (mass fraction of GM-material).

The absolute Limit of Quantification (LOQ_{abs}) has been determined according to the ENGL guidance document "Verification of analytical methods for GMO testing when implementing interlaboratory validated methods - Version 2" by a dilution series of five levels of target DNA (CSP-428), using a uniform concentration of non-target DNA (salmon sperm DNA 20ng/µl) for each dilution level. For each dilution level, 12 PCR replicate measurements were performed.

The LOQ_{abs} is estimated as the last dilution level where the RSDr of the copy number measurements is below 25 %. A standard curve covering the range from 2500 to 40 genomic copies were included in the analysis. Results are shown in Table 12.

Table 12: result of the LOQ_{abs} determination

Nominal copy number	mean copy number	SD copy number	RSDr copy number (%)
**40**	42,71	9,24	21,6
**20**	25,26	7,28	28,8
**10**	11,16	5,30	47,5
**5**	5,91	2,81	47,5
**3**	2,41	4,27	50,3
**1**	2,01	1,62	80,7

The results in Table 12 indicate a value of 40 genomic copies for the LOQ_{abs} which is associated with a RSDr of 21,6%.

4 REFERENCES

Federal Office of Consumer Protection and Food Safety [Bundesamt für Verbraucherschutz und Lebensmittelsicherheit (BVL)]. Guidelines for the single-laboratory validation of qualitative real-time PCR methods. (2016).

European Network of GMO Laboratories (ENGL). Definition of minimum performance requirements for analytical methods of GMO testing. (2015).

European Network of GMO Laboratories (ENGL) "Verification of analytical methods for GMO testing when implementing interlaboratory validated methods - Version 2".(2017)

Bennett MD, Leitch IJ. Plant DNA C-values database (release 6.0, Dec. 2012). http://data.kew.org/cvalues

Uhlig, S. et al. Validation of qualitative PCR methods on the basis of mathematical–statistical modelling of the probability of detection. Accredit. Qual. Assur. 20, 75–83 (2015).
